# Non-thermal atmospheric-pressure plasma promotes cellulase production in *Neurospora crassa*

**DOI:** 10.1186/s40643-025-01006-z

**Published:** 2026-01-27

**Authors:** Nan-Nan Yu, Wirinthip Ketya, Kirubel Amsalu, Jun-Sup Lim, Hu-Nan Sun, Eun-Ha Choi, Gyungsoon Park

**Affiliations:** 1 Plasma Bioscience Research Center, Department of Plasma-Bio Display, Kwangwoon University, Seoul, 01897 Korea; 2 College of Life Science and Technology, Heilongjiang Bayi Agricultural University, Daqing, 163319 Heilongjiang P.R. China; 3 Department of Electrical and Biological Physics, Kwangwoon University, 20 Kwangwoon-ro, Nowon-gu, Seoul, 01897 Korea

**Keywords:** Micro-surface dielectric barrier discharge plasma, Cellulase production, Neurospora crassa, Calcium ion, Nitric oxide

## Abstract

Microorganisms, particularly filamentous fungi, have become the dominant platforms for industrial enzyme production due to their rapid growth, low cost, and adaptability. However, current production technologies face limitations in yield and cost-efficiency, prompting the need for innovative enhancement strategies. Non-thermal atmospheric-pressure plasma has emerged as a promising tool for stimulating microbial enzyme production. In this study, we have employed micro-surface dielectric barrier discharge (MS-DBD) plasma, which operates in a completely different manner from jet plasma, and evaluated its potential for enhancing the production of cellulolytic enzymes in *Neurospora crassa*. The extracellular activity of cellulases increased (maximum 10.41 ± 3.44% increase) after MS-DBD plasma treatment. The transcription levels of the four cellulase genes were significantly elevated (highest in the 120 s treatment). The fungal hyphal membrane was depolarized and chemically altered after plasma treatment. The levels of intracellular Ca^2+^ and nitric oxide (NO) were elevated, and a high-affinity Ca^2+^ influx system was activated after plasma treatment. Ca^2+^ channel inhibitors reduced fungal cellulase production by downregulating intracellular NO levels. Plasma-mediated enhancement of enzyme production seemed to occur at plasma energies below 500–600 J. However, the combination of the plasma source type and treatment time can affect the efficiency of enzyme production. We also observed the promotion of fungal cellulase production when jet plasma was applied to larger volume of fungal hyphae. Our results suggest that plasma may be a genetically and environmentally safe tool for fungal enzyme production on an industrial scale and can be applied to bioreactors.

**Graphical Abstract:**

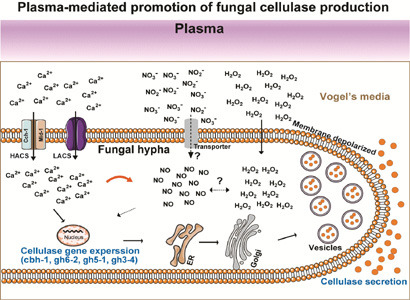

**Supplementary Information:**

The online version contains supplementary material available at 10.1186/s40643-025-01006-z.

## Introduction

According to a research report by MarketsandMarkets Blog, the global enzyme market is estimated to be valued at USD 14.0 billion in 2024 and is projected to reach USD 20.4 billion by 2029, with a compound annual growth rate of 7.8% (Hanniet et al. 2025). The expansion of economic investment indicates that market demand for industrial enzymes is constantly increasing. Among industrial enzymes, cellulases are one of the most commercially significant enzyme groups, ranking third in the global enzyme market (Bhardwaj et al. 2021). Cellulases are a complex mixture of enzymes that synergistically hydrolyze cellulose, the most abundant renewable biopolymer on Earth, into fermentable sugars. These enzymes have extensive applications in various industries, including biofuel production from lignocellulosic biomass, textile processing, paper and pulp manufacturing, animal feed enhancement, food and beverage processing, and detergent formulation (Kuhad et al. 2011). The cellulase system typically comprises three major enzyme classes: endoglucanases (EG) that randomly cleave internal β-1,4-glycosidic bonds, cellobiohydrolases (CBH) that processively release cellobiose from chain ends, and β-glucosidases (BG) that convert cellobiose to glucose (Ejaz et al. 2021). The efficiency of cellulose degradation depends on the synergistic action of these enzymes, making the optimization of cellulase production economically critical for industrial bioconversion processes. However, the current cellulase production technology is unable to meet the huge market demand and is accompanied by high production costs (Li et al. 2012). Therefore, researchers have explored various approaches to address supply and demand issues and minimize production costs. For example, the traditional method is to increase the transcription and secretion levels of industrial enzymes in genetically engineered strains using gene-editing technology or by adding inducers (Kant Bhatia et al. 2021). Although these approaches are feasible, several issues remain to be explored, including the safety and sustainability of genetically engineered strains, induction costs, and residue removal.

Non-thermal atmospheric-pressure plasma is a potential tool for solving problems associated with industrial enzyme production using microorganisms (Farasat et al. 2018; Kabarkouhi et al. 2024; Li et al. 2025; Veerana et al. 2021; Yu et al. 2022). Plasma is often referred to as the fourth state of matter along with solids, liquids, and gases (Langmuir 1928; Tonks 1967). Gas molecules are ionized in the plasma state, producing a mixture of positively charged ions, free energetic electrons (reactive species, free radicals), and neutral particles (Flannery 1983). Plasma is generally considered an environmentally friendly tool because it does not require the use of harmful chemicals (only ions are generated by ionized air) (Ekezie et al. 2017). This characteristic aligns with the growing demand for sustainable and environmentally friendly production processes. Compared to other strategies to promote enzyme production, plasma has great advantages in industrial enzyme production. Plasma processing equipment can be simply constructed, and the gases used for plasma generation (such as air) are relatively low-cost, thus minimizing the overall production costs. Plasma processing is additionally highly scalable and can be adapted to different production scales (from small- to large-scale production of microbial enzymes).

Although plasma shows great application potential for industrial enzyme production, several issues still need to be further explored, including accumulating more evidence on plasma-mediated enhancement of enzyme production using various microbial species, plasma devices, and feeding gases; elevating the efficiency of and yield of enzyme production; elucidating the underlying mechanisms for plasma-mediated production of microbial enzymes; and optimizing plasma equipment, processing conditions, and microbial species (type of plasma device, treated objects, and distance). *Neurospora crassa* is a model filamentous fungus and a natural producer of cellulases, which represent the third most widely used industrial enzymes (Waters et al. 2017). Previous studies have shown that jet plasma treatment can promote extracellular cellulase production in *N. crassa* (Yu et al. 2022). To investigate whether different types of plasma have the same effect on fungal cellulase production, we used MS-DBD plasma, another plasma type, and analyzed the effects on enzyme production in *N. crassa* in this study. Furthermore, we explored the underlying mechanisms, focusing on Ca^2+^ and NO involved regulations. The findings provide experimental evidence to establish a database for a solid theoretical foundation for future industrial applications of plasma technology.

## Materials and methods

### Plasma devices

**Fig. 1:**
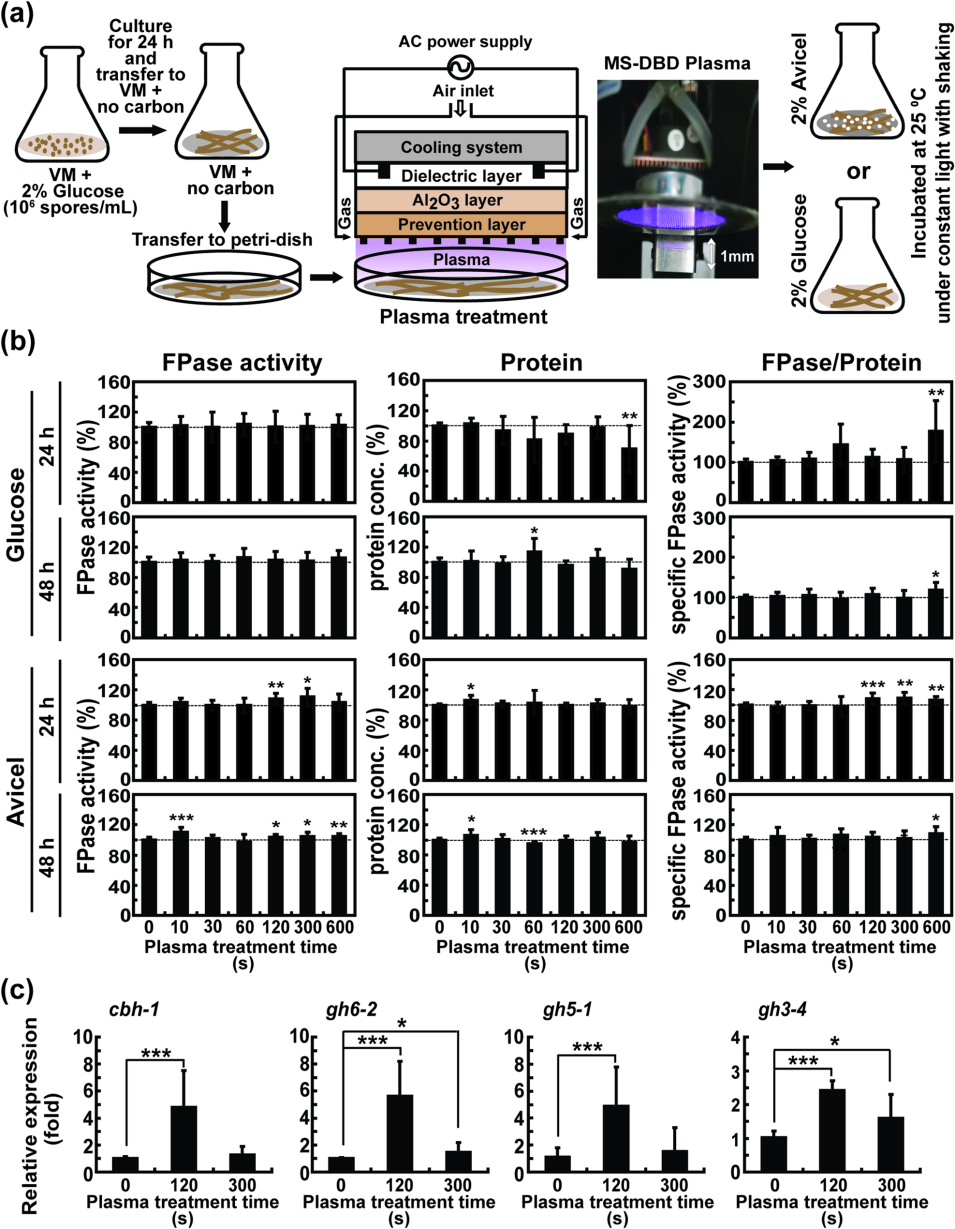
Effect of MS-DBD plasma treatment on the production of fungal cellulolytic enzymes. **a** Experimental scheme for plane-type MS-DBD plasma treatment on *N. crassa* hyphae in VM media. **b** Relative filter paper enzyme (FPase) activity (total activity of cellulolytic enzymes), relative total protein concentration, and relative specific FPase activity (total enzyme activity divided by total protein) in avicel or glucose media measured at 24 and 48 h after MS*-*DBD plasma treatment. The dashed line indicates the 100% level. **c** Level of mRNA of four cellulolytic enzymes (*cbh1*, *gh6-2*, *gh5-1*, and *gh3-4*) in fungal hyphae grown in avicel medium at 4 h after MS-DBD plasma treatment. Each value is the mean of six or nine replicate measurements three independent biological replicates and two-three technical replicates): * *p* < 0.05, ** *p* < 0.01, *** *p* < 0.001 as determined by Student’s *t*-test (control vs. treatment)

The main plasma device used in this study was an micro-surface dielectric barrier discharge (MS-DBD) plasma, as described by Ji et al. (2019) (Fig. 1a). Plasma was generated using an electric power of 1.2 kV input voltage and 50–63 mA current using air as a feeding gas with 1.5 L/min flow rate. A plasma jet device (Supplementary Figure S1) that was newly constructed was also used with different electric powers (2.1–9.4 W) in several experiments. The configuration of the plasma jet device was as follows: a dielectric glass tube containing a needle electrode was placed inside a ground metal electrode with a gap of 1 mm between the dielectric glass end and the tip of the ground metal electrode (Supplementary Figure S1). All plasma devices were provided by the Plasma Bioscience Research Center at Kwangwoon University (Seoul, Korea). Air was supplied to the plasma devices at 1.5 L/min to generate plasma.

A high-voltage probe was connected to a high-voltage electrode (Tektronix, Beaverton, OR, USA), and a current probe (LeCroy, Chestnut Ridge, NY, USA) was connected to the ground electrode to measure the voltage and current during plasma generation. During the plasma discharge, the current and voltage were measured using an oscilloscope (LeCroy). The emission spectra of different reactive species were monitored using a spectrometer (HR4000, Ocean Optics, Dunedin, FL, USA).

The energy during plasma discharge can be calculated using Eq. (1), where t_1_ and t_2_ are the initial and final time durations for one complete cycle, respectively. The duty ratio (DR) of the plasma was determined using Eq. (2). Equation (3) was used to evaluate the dissipated power (P_diss_).

1 $$\:\mathrm{E}\mathrm{n}\mathrm{e}\mathrm{r}\mathrm{g}\mathrm{y}\:\left(\mathrm{J}\right)=\:{\int\:}_{{\mathrm{t}}_{1}}^{{\mathrm{t}}_{1}}\mathrm{V}\left(\mathrm{t}\right)\times\:\mathrm{I}\left(\mathrm{t}\right)\:\mathrm{d}\mathrm{t}$$

2 $$\:\mathrm{D}\mathrm{u}\mathrm{t}\mathrm{y}\:\mathrm{r}\mathrm{a}\mathrm{t}\mathrm{i}\mathrm{o}\:\left(\mathrm{\%}\right)=\:\frac{\mathrm{o}\mathrm{n}\:\mathrm{t}\mathrm{i}\mathrm{m}\mathrm{e}}{\mathrm{o}\mathrm{n}\:\mathrm{t}\mathrm{i}\mathrm{m}\mathrm{e}+\mathrm{o}\mathrm{f}\mathrm{f}\:\mathrm{t}\mathrm{i}\mathrm{m}\mathrm{e}}\times\:100$$

3 $$\:{\mathrm{P}}_{\mathrm{d}\mathrm{i}\mathrm{s}\mathrm{s}}\left(\mathrm{W}\right)=\mathrm{F}\mathrm{r}\mathrm{e}\mathrm{q}\mathrm{u}\mathrm{e}\mathrm{n}\mathrm{c}\mathrm{y}\times\:\mathrm{d}\mathrm{u}\mathrm{t}\mathrm{y}\:\mathrm{r}\mathrm{a}\mathrm{t}\mathrm{i}\mathrm{o}\times\:Energy$$

## Fungal strains and plasma treatment

*N. crassa* strains, FGSC 4200 (wild type, genotype ORS-SL6a, mating type mat a) and FGSC 11,707 (knockout mutant of *mid-1*, genotype Δ*mid-1*/NCU06703, mating type mat a), obtained from Fungal Genetics Stock Center (FGSC, Manhattan, KS, USA), were used in this study. FGSC 4200 strain was maintained on Vogel’s minimal (VM) agar, and FGSC 11,707 strain on VM agar medium supplemented with 200 µg/mL hygromycin (Calbiochem, San Diego, CA, USA). To obtain spores, the fungus was inoculated onto VM agar media in a flask and cultured at 30 °C in darkness for two days and then at 25 °C in light for 12 days. Sterile deionized (DI) water (50 mL) was added to the flask, which was vigorously shaken. The fungal suspension was filtered through three layers of sterile Miracloth (EMD Millipore, Burlington, MA, USA), and the spore suspension was centrifuged at 3,134 ×*g* for 5 min. After discarding the supernatant, the pellet was resuspended in sterile DI water.

A spore suspension of *N. crassa* was inoculated into 30 mL of VM containing 2% (w/v) glucose (1 × 10^6^ spores/mL) and placed in a glass Erlenmeyer flask (85 mm diameter, 140 mm height, 34 mm neck diameter). The flask was incubated at 25 °C with shaking (200 rpm) under constant light for 24 h. Fungal mycelia were then collected by filtration through two layers of Miracloth (EMD Millipore) and washed with deionized water. Fungal hyphae were suspended in 25 mL fresh VM medium without a carbon source, and the suspension was transferred to a 90 mm petri-dish (Fig. 1). MS-DBD plasma was then applied to fungal hyphae in a petri-dish, as presented in Fig. 1a. After the plasma treatment, the fungal hyphae were transferred to a flask containing VM without a carbon source, and 5 mL of 12% (w/v) glucose (Duksan, Seoul, South Korea) or avicel (Avicel PH-101, Sigma-Aldrich, St. Louis, MI, USA) was added to each flask to obtain a final concentration of 2% (w/v) (Fig. 1a). Flasks were incubated at 25 °C with shaking (200 rpm) under constant light for the indicated time.

## Determining the activity of cellulolytic enzymes and the concentration of total protein

The production of cellulases by *N. crassa* was assessed by measuring cellulolytic activity and total protein concentration in the media. Fungal hyphae were treated with plasma and transferred to induction (avicel in media) or no-induction (glucose in media) media, as described earlier. After incubation for 24 and 48 h, the culture media were harvested and centrifuged at 2390 × *g* for 10 min to remove the hyphae. The supernatant was stored at 4 °C until further analyses. Cellulolytic activity in the culture supernatant was measured by determining the rate of filter paper degradation (FPase) by enzymes in the culture supernatant, as described previously (Yu et al. 2022). The total protein concentration in the culture supernatant was measured using a Bradford protein assay kit (Bio-Rad, Hercules, CA, USA).

To measure FPase activity, a reaction mixture containing Whatman filter paper no. 1 (substrate; 2.2 mg filter paper), 30 µL of 0.1 M acetate buffer (pH 5.6), and 30 µL of culture supernatant was incubated at 50 °C for 30 min. The level of liberated reducing sugars (the product of the enzymatic reaction) was measured by adding 120 µL of 3,5-dinitrosalicylic acid (DNS; Sigma-Aldrich) into a reaction mixture and then boiling the reaction mixture for 10 min. Finally, 720 µL of deionized water was added to the reaction mixture, and the solution absorbance was measured at 540 nm using a microplate reader (BioTek, Winooski, VT, USA). The amounts of the products (reducing sugars) were calculated using a maltose standard curve. Enzyme activity is indicated in international units (IU), where one IU is defined as the amount of enzyme that can produce 1 µM of reducing sugars (product) per min.

To measure the protein concentration, 10 µL of culture supernatant was placed in each well of a 96-well plate, and 200 µL of Bradford solution was added to each well. After incubation for 5 min at 25 °C in the dark, the solution absorbance was measured at 595 nm using a microplate reader (BioTek).

Specific FPase activity was calculated by dividing the FPase activity by protein concentration. The relative percentages (%) of FPase activity, protein concentration, and specific FPase activity of the plasma-treated sample compared to those of the control (untreated sample) were calculated as follows: (FPase activity, protein concentration, or specific FPase activity of the plasma-treated sample/average value of FPase activity, protein concentration, or specific FPase activity of the control) × 100.

## Analysis for membrane potential and levels of intracellular NO and Ca^2+^

The membrane potential and levels of intracellular Ca^2+^ and NO were analyzed as previously described (Yu et al. 2023). Fungal mycelia were harvested at the indicated incubation times after plasma treatment, as described in earlier section. The harvested fungal mycelia were washed twice with 1× phosphate-buffered saline (PBS). For detecting membrane potential, fungal mycelia were placed in 500 µL of 50 µg/mL bis-(1,3-dibutylbarbituric acid) trimethine oxonol (DiBAC4(3); Invitrogen, Carlsbad, CA, USA) and incubated at 4 °C in the dark for 1 h. For detecting intracellular Ca^2+^, fungal mycelia were placed in 500 µL of 10 µM Fluo3-AM (Invitrogen) and incubated at 25 °C in the dark for 1 h. For detecting intracellular NO, fungal mycelia were placed in 500 µL of 20 µM 4-amino-5-methylamino-2′,7′-difluorofluorescein diacetate (DAF-FM DA, Thermo Fisher, Waltham, MA, USA) and incubated at 25 °C for 1 h. After incubation, the fungal mycelia were washed with 1× PBS at least three times and examined under an FV-100 MPE confocal laser scanning microscope (Olympus Corporation, Tokyo, Japan).

## cPTIO, SNP, and LaCl_3_ treatment

After plasma treatment, the effect of scavenging of intracellular nitric oxide (NO), addition of NO, or inhibition of Ca^2+^ channel on enzyme production was examined using NO scavenger (cPTIO: 2-(4-carboxyphenyl)-4,5-dihydro-4,4,5,5-tetramethyl-1 H-imidazolyl-1-oxy-3-oxide), NO donor (SNP: sodium nitroprusside), or Ca^2+^ channel blocker (LaCl_3_: lanthanum(III) chloride). As presented in Fig. 1a, N. *crassa* spores were inoculated into 30 mL of VM liquid containing 2% (w/v) glucose (1 × 10^6^ spores/mL). After incubation for 24 h, the fungal mycelia were recovered and resuspended in VM liquid without a carbon source. The fungal mycelial suspension was exposed to MS-DBD plasma for 0, 120, or 300 s, and avicel (final conc. 2%), supplemented with cPTIO (final conc. 10 mM: Calbiochem, San Diego, CA, USA), SNP (final conc. 0.1 mM: Sigma-Aldrich), or LaCl_3_ (final conc. 5 mM: Sigma-Aldrich) was added immediately to the suspension after the plasma treatment.

Measurement of pH, oxidation-reduction potential (ORP), electrical conductivity (EC),and levels of hydrogen peroxide (H_2_O_2_) and nitrogen oxides (NOx; NO, NO_***2***_^−^, NO_3_^−^) in media.

VM media without fungal hyphae was treated with plasma and then analyzed. The EC, ORP, and pH of treated media were measured using a PCTSTestr™ 50 Waterproof Pocket pH/Cond/TDS/Salinity Tester (Oakton Instruments, Vernon Hills, IL, USA), an ExStikTM Model RE300 waterproof ORP meter (Extech, Nashua, NH, USA), and a portable pH meter (Oakton Instruments), respectively. H_2_O_2_ and NOx levels in media were measured using an AmplexTM Red Hydrogen Peroxide/Peroxidase Assay Kit (Molecular Probes, Eugene, OR, USA) and QuantiChromTM Nitric Oxide Assay Kit (BioAssay Systems, Hayward, CA, USA), respectively, according to the manufacturer’s instructions.

### Quantitative real-time PCR analysis and protein gel electrophoresis

Fungal mycelia were collected at the indicated incubation times after the plasma treatment, and immediately frozen and ground in liquid nitrogen. The total RNA was extracted from the ground mycelia powder using RNAiso Plus (TaKaRa Bio, Shiga, Japan), and cDNA was synthesized using ReverTra Ace qPCR RT Master Mix with gDNA Remover (Toyobo, Osaka, Japan) following the manufacturer’s protocol. Real-time PCR was performed using iQ SYBR Green Supermix (Bio-Rad) and a CFX 96TM Real Time Instrument (Bio-Rad) following the manufacturer’s instructions. The mRNA levels for each enzyme were normalized to the reference gene (β-actin) and determined as follows: mRNA level of enzyme gene = 2^−∆∆Ct^, where ∆∆Ct = (Ct _target_ − Ct _reference_)_plasma treatment_ − (Ct _target_ − Ct _reference_)_control_ (Livak and Schmittgen 2001). The primer sequences are listed in Table 1.

**Table 1:** List of primers used in qRT-PCR

Genes	Primer sequences
*β-actin*	Forward- 5′-TGA TCT TAC CGA CTA CCT-3′
	Reverse- 5′-CAG AGC TTC TCC TTG ATG-3′
*cbh-1*	Forward- 5′-ATC TGG GAA GCG AAC AAA G-3′
	Reverse- 5′-TAG CGG TCG TCG GAA TAG-3′
*gh6-2*	Forward- 5′-CCC ATC ACC ACT ACT ACC-3′
	Reverse- 5′-CCA GCC CTG AAC ACC AAG-3′
*gh5-1*	Forward- 5′-GAG TTC ACA TTC CCT GAC A-3′
	Reverse- 5′-CGA AGC CAA CAC GGA AGA-3′
*gh3-4*	Forward- 5′-AAC AAG GTC AAC GGT ACG TGG-3′
	Reverse- 5′-TCG TCA TAT CCA TAC CAC TGT TTG-3′
*nit-2*	Forward- 5′-CGAACAAGCAGTCCGATCACCAG-3′
	Reverse- 5′-GCCACCATCCTCCTCGTCTCC-3′
*nit-3*	Forward- 5′-AACGACCTCGCCAGCACTC-3′ Reverse- 5′-TGTTGGTGTTGGTGTTGGATGAG-3′
*nit-4*	Forward- 5′-GCAACAGCAACAGCAACAGCAG-3′ Reverse- 5′-TCCACCTCCTACCGTCGTCATTC-3′
*nit-6*	Forward- 5′-CAGGAGCGGTTCAAGCAGTTC-3′ Reverse- 5′-GCCACATCACGGGTCTTTCTTG-3′
*nit-10*	Forward- 5′-TGACGACGACGACGAAGATGG-3′ Reverse- 5′-GAATGGTGGTGGTGTTGGAAGG-3′

For protein gel electrophoresis, fungal mycelia were removed by centrifugation, and culture supernatants were collected 24 and 48 h after plasma treatment, as described in earlier section. Sample buffer (5×) was added to the culture supernatant, and the mixture was boiled for 5 min. The sample (20 µL or 3 µg) was loaded onto a sodium dodecyl sulfate (SDS)-polyacrylamide (12%) gel. The gel was run at 140 V for approximately 1 h and 30 min and then stained overnight using Coomassie blue R-250 (Bio-Rad) for 12 h. The gel was then washed using the destain solution to remove the background stain. Finally, the gel was imaged using a ChemiDocTM MP imaging system (Bio-Rad) and analyzed using ImageJ software version 1.52a (National Institute of Health, Bethesda, MD, USA).

## Analysis for Raman spectroscopy

Fungal mycelia were treated with plasma for 300 s, as described in earlier section, and the hyphae were harvested immediately after treatment. Harvested fungal mycelia were washed with 1× PBS and mounted on glass slides for Raman spectroscopy. The samples were examined using a Raman microscope (WITec alpha300, Oxford Instruments, Abingdon-on-Thames, England) with an excitation wavelength of 488 nm at 50× magnification. The laser power was fixed at 3 mW, and the integration time was maintained at 5 s, with 15 accumulations. Each measurement was repeated three times to identify the noise calibrated by smoothing and baseline correction.

### Statistical analysis

All data are presented as the mean ± standard deviation (SD) from at least six replicates. A paired Student’s t-test and two-way analysis of variance were performed, followed by Tukey’s post-hoc test. A *p*-value < 0.05 was considered statistically significant. SPSS Statistics Software version 25 (IBM, Chicago, IL, USA) was used for statistical analysis.

## Results

### MS-DBD plasma treatment can enhance the production of cellulases in N. crassa

The voltage and current profiles during the discharge of the MS-DBD plasma (Fig. 1a) are described in a previous study (Ji et al. 2019). Plasma was generated with 1.2 kV input voltage and 62 mA current using 1.5 L/min air as the feeding gas (Ji et al. 2019). The optical emission spectra (OES) of the plasma showed excited nitrogen species corresponding to the nitrogen second positive system (Ji et al. 2019).

Generally, cellulase production is induced by cellulosic materials in the media (Oguntimein et al. 1992). We analyzed enzyme activity under non-induction (glucose-containing media) and induction (avicel; cellulose powder in the media) conditions. In media containing glucose (non-induction conditions), cellulolytic activity, measured as FPase activity (total activity of enzymes degrading cellulose in filter paper), was not significantly different between untreated and MS-DBD plasma-treated samples (Fig. 1b and Supplementary Table S1). We measured FPase activity as an indicator of overall cellulolytic capacity, which directly reflects the practical degradation efficiency for industrial cellulose bioconversion. The total protein level detected in the medium after 24 h was slightly reduced upon plasma treatment, and no significant change was observed after 48 h (Fig. 1b and Supplementary Table S1). In media containing avicel (induction condition), FPase activity significantly increased 24 and 48 h after MS-DBD plasma treatment for 120–600 s (Fig. 1b and Supplementary Table S1). After 24 h of incubation, FPase activity in the media increased up to 10.41% in the plasma-treated sample (plasma treatment for 300 s) (Fig. 1b and Supplementary Table S1). The total protein concentration in the media after 24 h was not significantly different between the non-plasma- and plasma-treated samples, except for a 10 s plasma-treated sample, which showed a 6.43% increase (Fig. 1b and Supplementary Table S1). The specific activity of FPase (total FPase activity divided by total protein amount) was significantly higher after plasma treatment for 120 s, 300 s (highest 9.03% increase), and 600 s (Fig. 1b and Supplementary Table S1). After 48 h of incubation, FPase activity in the media was significantly higher in samples treated with plasma for 10 (highest 10.36 ± 2.08% increase), 120, 300, and 600 s than in the control (no plasma) sample (Fig. 1b and Supplementary Table S1). The total protein concentration in the media was significantly higher in samples treated with plasma for 10 s (the highest increase of 6.30 ± 2.30%) (Fig. 1b and Supplementary Table S1). The specific activity of FPase was significantly higher in the 600 s plasma-treated sample (8.63 ± 3.03% increase) (Fig. 1b and Supplementary Table S1).

We also investigated whether enhanced cellulase activity in media resulted from increased levels of four enzymes, two cellobiohydrolases (*cbh1* and *gh6-2*), an endoglucanase (*gh5-1*), and a β-glucosidase (*gh3-4*), which might be secreted into media and be involved in the degradation of filter paper (Phillips et al. 2011). The transcription levels of genes encoding the four cellulolytic enzymes significantly increased when the fungi were treated with plasma for 120 s (Fig. 1c). The mRNA levels of *gh6-2* and *gh3-4* in the plasma-treated fungal cells for 300 s were significantly higher than those in the control sample (Fig. 1c). The levels of cellulolytic enzymes secreted into the culture medium were assessed by profiling the secreted proteins in the medium using SDS-PAGE. When the same volume of culture medium (20 µL) or the same amount of proteins (3 µg) from each treatment was loaded, no obvious difference in the intensity of the four enzyme protein bands was observed between the no plasma and plasma-treated samples after incubation for 24 and 48 h (Supplementary Figure S2).

### MS-DBD plasma treatment can change the physical and chemical properties of the culture medium

**Fig. 2:**
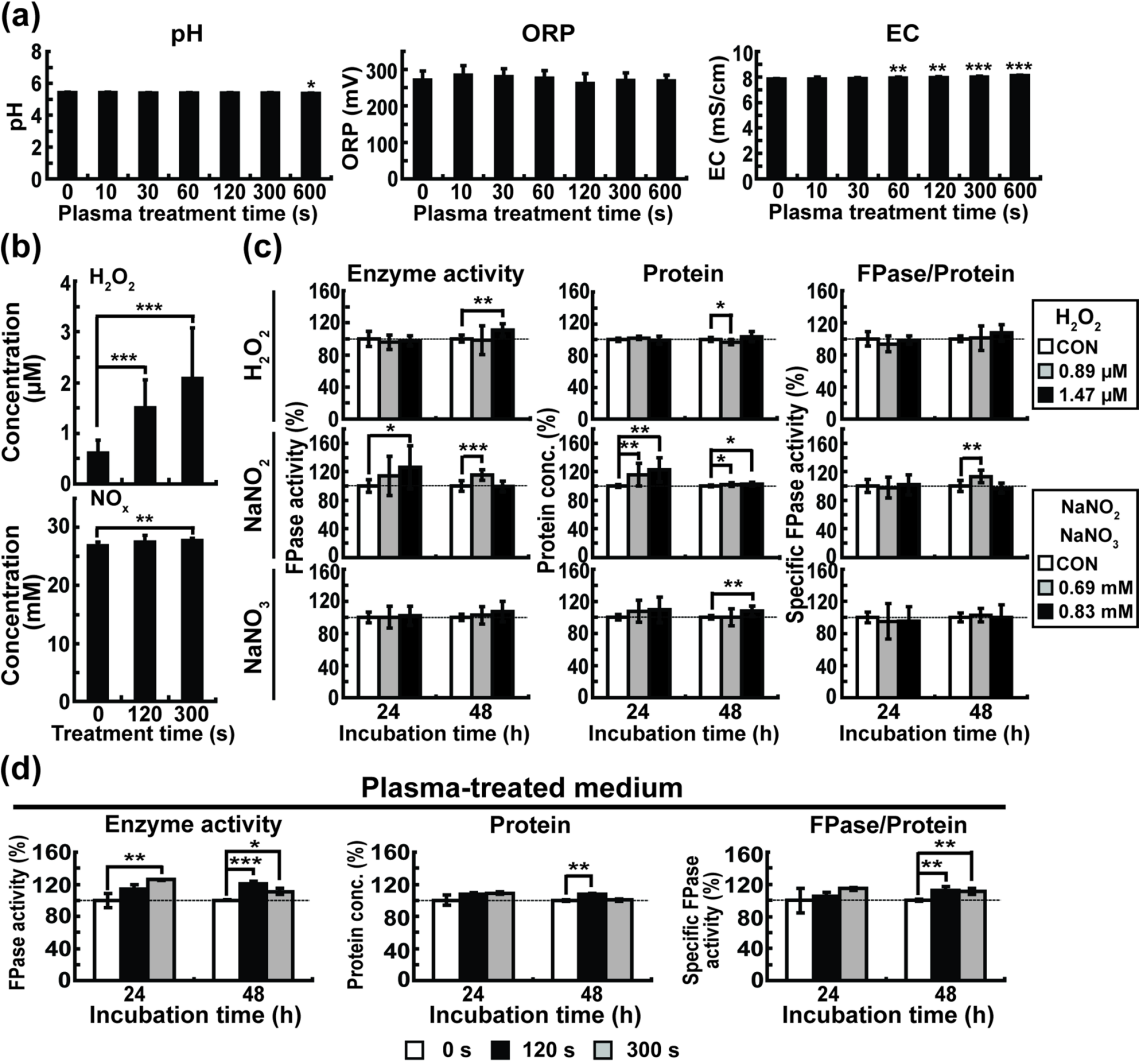
Effect of MS-DBD plasma treatment on culture media. **a** pH, ORP, and EC of VM medium without carbon at 0 h after MS*-*DBD plasma treatment. **b** The concentration of H_2_O_2_ and NO_X_ (NO + NO_2_^−^ + NO_3_^−^) in VM medium (no carbon) at 0 h after MS*-*DBD plasma treatment. **c** Relative filter paper enzyme activity, relative total protein concentration, and relative specific cellulolytic enzyme activity in avicel medium supplemented with H_2_O_2_, NaNO_2,_ or NaNO_3_. **d** Relative filter paper enzyme activity, relative total protein concentration, and relative specific cellulolytic enzyme activity in medium after fungus was incubated in plasma-treated avicel medium (treated for 0, 120, and 300 s). Each value is the mean of three or twelve replicate measurements (three independent biological replicates and two-four technical replicates): * *p* < 0.05, ** *p* < 0.01, *** *p* < 0.001 as determined by Student’s *t*-test (control vs. treatment)

To determine whether plasma changes the physicochemical properties of the culture media, we measured the pH, EC, and ORP of the VM medium immediately after plasma treatment. There was no dramatic change in the pH, ORP, or EC of the medium after plasma treatment, although the pH was very slightly reduced and the EC increased after plasma treatment for a certain amount of treatment time (Fig. 2a). After plasma was applied to the media for 120 s and 300 s, the concentration of H_2_O_2_ increased from 0.61 µM (0 s) to 1.50 µM (120 s) and 2.09 µM (300 s), respectively (Fig. 2b). NO_X_ (NO, NO_2_^−^, and NO_3_^−^) levels in culture media increased from 26.82 mM (0 s) to 27.51 mM (120 s) and 27.66 mM (300 s), respectively (Fig. 2b). We further explored whether the changes in NO_X_ and H_2_O_2_ concentrations were related to fungal cellulase production. We treated the fungi with different concentrations of H_2_O_2_, NO_2_^−^, or NO_3_^−^, using chemicals. Our results showed that the total protein concentration increased significantly in all treated groups compared to that in the untreated group. FPase activity increased only in the H_2_O_2_ and NO_2_^−^-treated groups, and specific enzyme activity increased only in the NO_2_^−^-treated group (Fig. 2c).

We treated the VM medium with plasma and cultured the fungi in the plasma-treated medium for 24 and 48 h. After fungi grew in the plasma-treated medium, the FPase activity, total protein concentration, and specific enzyme activity were significantly higher than those in the untreated medium, mostly after 48 h of incubation (Fig. 2d and Supplementary Table S2).

### MS-DBD plasma induces cell membrane depolarization and chemical changes

**Fig. 3:**
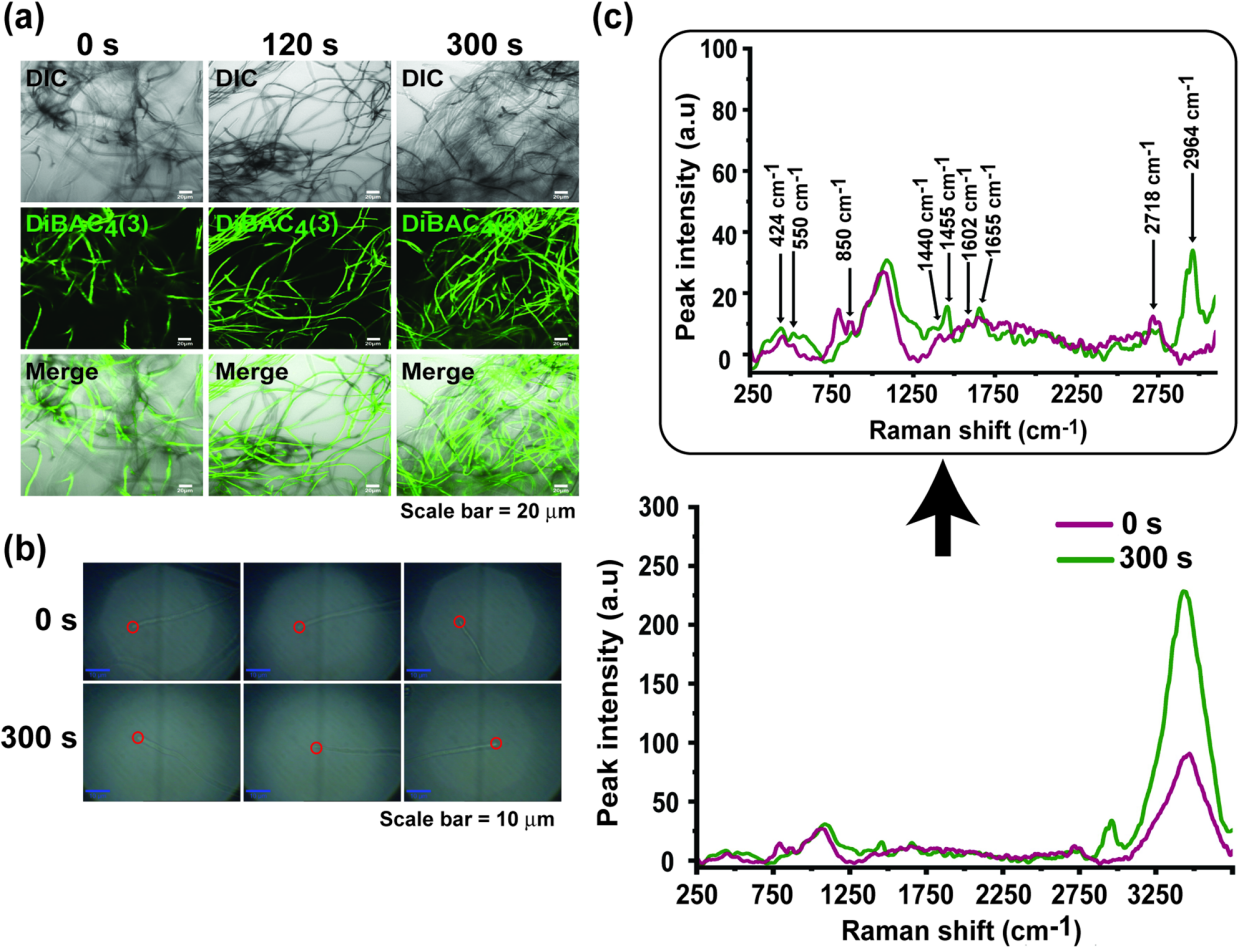
Effects of MS-DBD plasma treatment on fungal cell membranes. **a** Membrane depolarization of *N. crassa* hyphae measured using DiBAC_4_(3). **b** The 488 nm laser was pointed at the tip of *N. crassa* hyphae during Raman spectroscopy analysis. Three different hyphal tips were scanned in each control and treated sample. **c** Raman spectra of plasma-treated (300 s) and untreated (0 s) fungal hyphae

The cell membranes of fungal hyphae were significantly depolarized immediately after plasma treatment (0 h of incubation) compared to those of untreated hyphae (Fig. 3a and Supplementary Figure S3a). After 24 h of incubation, the difference in depolarization between the plasma-treated and untreated samples slightly diminished (Supplementary Figure S3b).

Raman spectral analysis was performed to examine the chemical changes in the components of the fungal cell membrane. The tips of several different fungal hyphae were scanned to acquire information from many areas (Fig. 3b). Raman spectral peaks detected on the tips of fungal hyphae in the plasma-treated (300 s) and untreated (0 s) samples are presented in Fig. 3c. Each Raman peak was analyzed in terms of wavelength, functional group bonding, and chemical identity (Table 2). The Raman peaks at 1655, 1602, 1455, and 1440 cm^− 1^ likely correspond to C = C stretching, C = C in-phase stretching of ergosterol, C-H bending, and C-H bending of the aliphatic chain included in the lipid spectra, respectively (Table 2; Fig. 3c) (Noothalapati et al. 2016). The level of peaks at 1655, 1455, and 1440 cm^− 1^ was slightly higher in plasma-treated than untreated fungal hyphae (Fig. 3c). Raman band at 850 cm^− 1^ seemed to be a tyrosine band from proteins in cytoplasm (Noothalapati et al. 2016), and no difference in peak level was observed between plasma-treated and untreated fungal hyphae (Table 2; Fig. 3c). Bands below 1000 cm^− 1^ indicating the presence of polysaccharides, such as those at 550 and 424 cm^− 1^ (Noothalapati et al. 2016), were more prominent in plasma-treated samples than in untreated samples (Table 2; Fig. 3c). Interestingly, a new peak at approximately 2964 cm^**−** 1^ was observed in the plasma-treated fungal hyphae, which is known to be the CH_3_ asymmetric stretching of *n*-pentane (Qiao and Zheng 2005). The broad spectral band beyond 2900 cm^**−** 1^ represents a typical O-H stretching mode from water molecules (Fig. 3c) (Durickovic 2016).

**Table 2:** Summary of different Raman peaks and their respective assignments (Qiao 2005; Noothalapati, 2016)

Raman shift (cm^− 1^)	Vibrational mode	Peak assignment
2964	CH_3_ asymmetric stretching	*n*-pentane
1655	C = C (Stretching)	
1602	C = C (in-phase stretching)	Ergosterol
1455	C-H (Bending)	–
1440	C-H (Bending)	Aliphatic chain
850		Tyrosine residue in protein
550		Glycosidic band in dextran
424	Skeletal vibration	Non-specific glucan

### MS-DBD plasma enhances the production of cellulases through accelerating Ca^2+^ influx

**Fig. 4:**
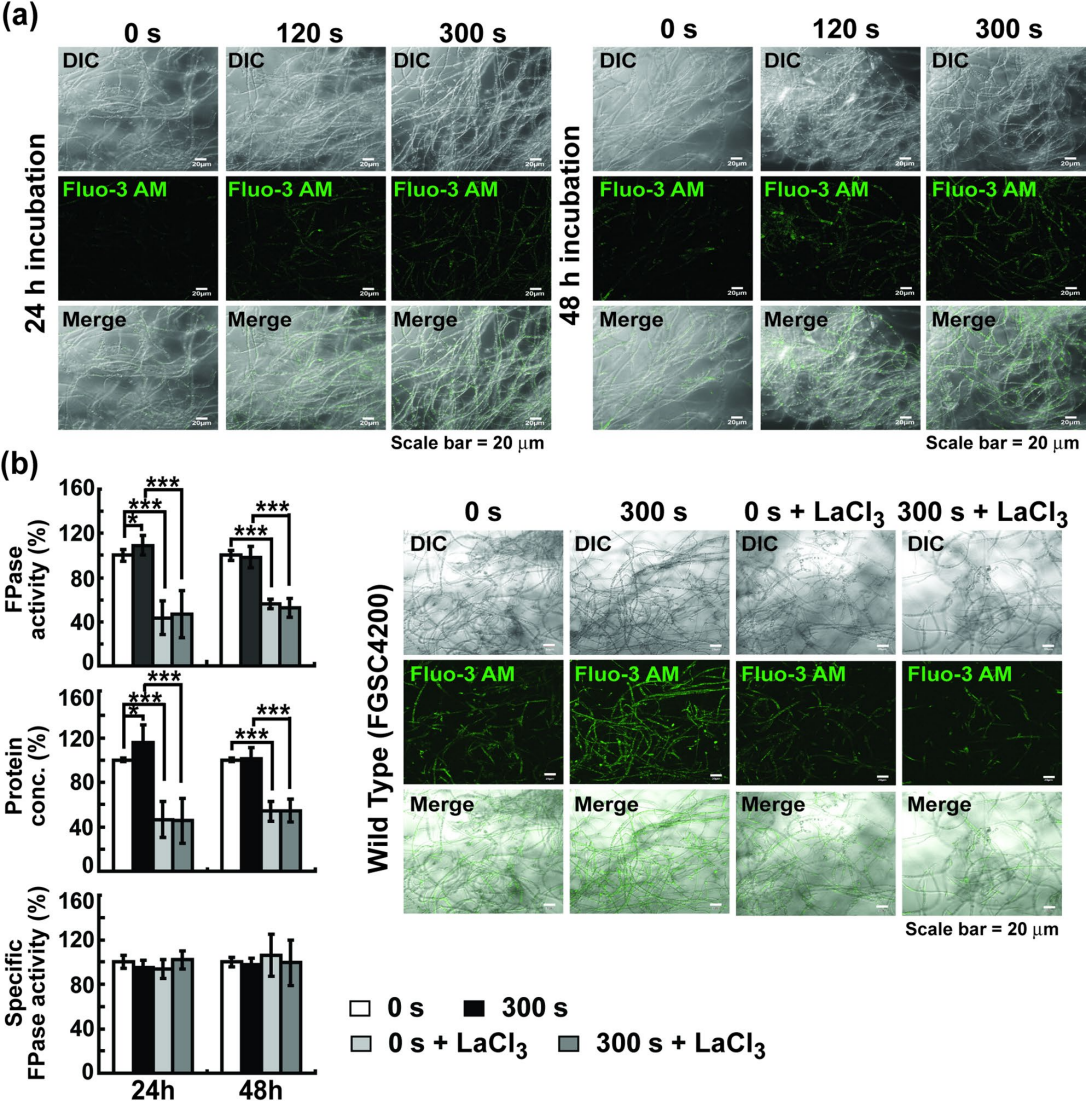
Analysis for intracellular Ca^2+^ accumulation after MS-DBD plasma treatment and its effect on cellulases production. **a** Intracellular Ca^2+^ was detected using a fluorescent dye (Fluo-3 AM) in MS-DBD plasma-treated fungal hyphae in avicel media after 24 h and 48 h of incubation. **b** Effect of LaC1_3_ (Ca^2+^ channel blocker, 5 mM) on cellulase production and Ca^2+^ accumulation in plasma-treated and untreated fungal hyphae grown in avicel media. DIC: differential interference contrast, Fluo-3 AM: fluorescence, Merge: combined image of DIC and fluorescence. Each value is the average of nine replicate measurements (three independent biological replicates and three technical replicates): * *p* < 0.05, *** *p* < 0.001 as determined by Student’s *t*-test

Because we previously observed that the expression of cellulase mRNAs is regulated by Ca^2+^ and NO (Yu et al. 2022), which are important intracellular secondary messengers during plasma stimulation (Kawase et al. 2020; Li et al. 2017), intracellular Ca^2+^ levels were assessed by fluorescent staining. The fluorescence intensity (an indicator of intracellular Ca^2+^) increased in plasma-treated fungal hyphae after incubation for 4, 24, and 48 h (Fig. 4a and Supplementary Figure S4). However, no difference in the fluorescence intensity was observed between 120 and 300 s plasma treatment (Fig. 4a and Supplementary Figure S4).

To further verify that plasma promoted fungal cellulase production by enhancing Ca^2+^ accumulation in cells, we inhibited Ca^2+^ influx by blocking Ca^2+^ channels on the cell membrane (LaCl_3_). When the Ca^2+^ channel blocker LaCl_3_ was applied to fungal hyphae, FPase activity and protein concentration in the media, as well as intracellular Ca^2+^ levels (fluorescence intensity), decreased in both untreated and treated plasma samples (Fig. 4b, Supplementary Table S3a, and Supplementary Figure S5). This indicates that plasma can activate Ca^2+^ channels in the cell membrane, but not Ca^2+^ stores (endoplasmic reticulum, Golgi apparatus, and vesicles). The mRNA levels of the two *plc* genes, which are signaling molecules that trigger the opening of channels on calcium storage membranes, did not change significantly after plasma treatment (Supplementary Figure S6).

MS-DBD plasma was also applied to the knockout mutant, *mid-1*, encoding a putative protein comprising a high-affinity Ca^2+^ influx system (HACS) that activates the downstream transcription factor crz1 for cellulase production (Liu et al. 2015; Muller et al. 2001; Randhawa et al. 2024). The deletion of *mid-1* inhibited the growth of fungal hyphae in VM (Supplementary Figure S7a). When the *mid-1* mutant was treated with MS-DBD plasma and grown in avicel media, the FPase activity and protein concentration in the media did not differ between the untreated and treated *mid-1* mutants after 24 and 48 h (Supplementary Figure S7b and Supplementary Table S3b). However, the intracellular Ca^2+^ concentration increased slightly in the *mid-1* mutant after MS-DBD plasma treatment (Supplementary Figure S7c).

### MS-DBD plasma-mediated enhancement of enzyme production may be related to endogenous generation of NO

**Fig. 5:**
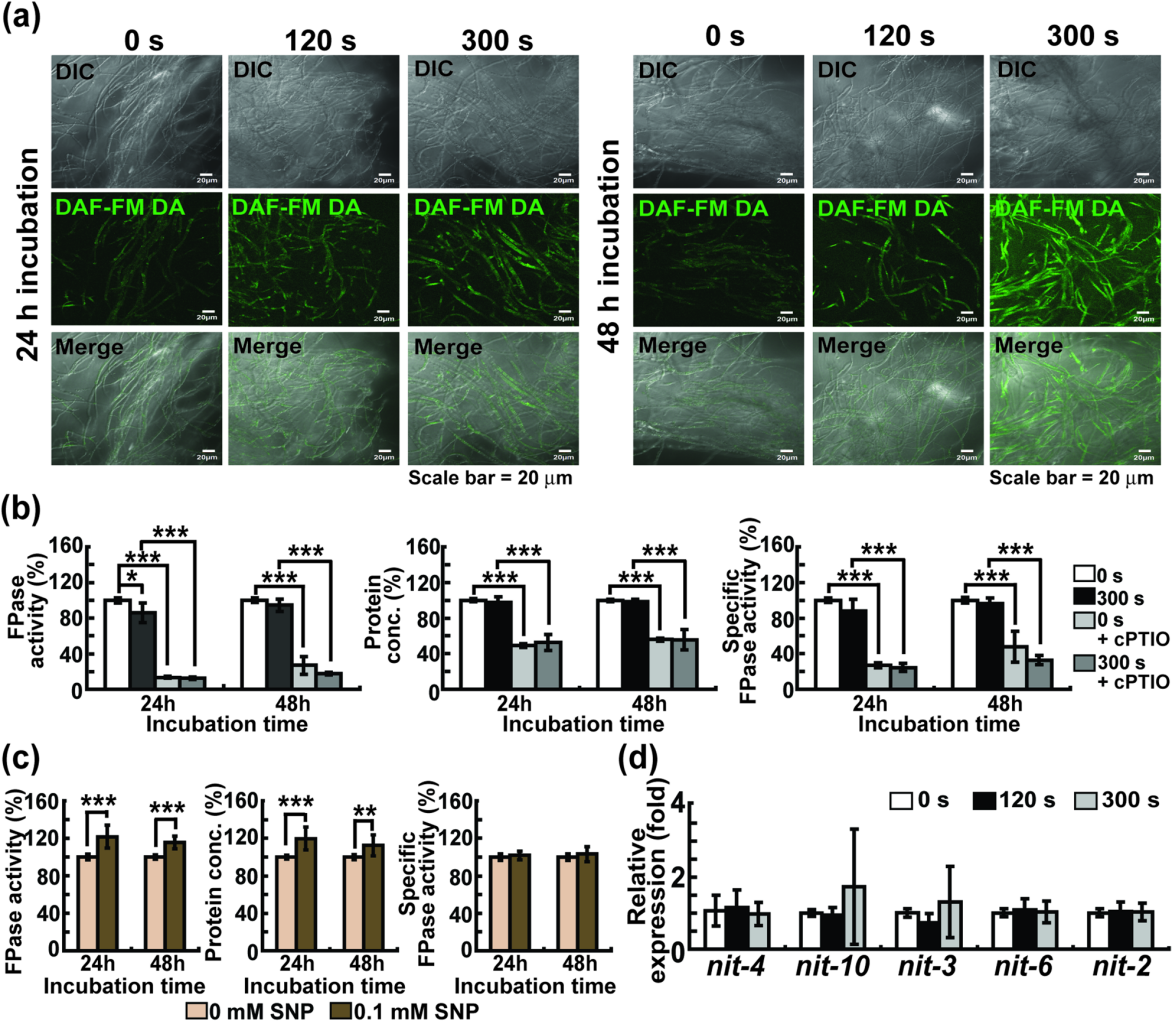
Intracellular NO production after MS-DBD plasma treatment. **a** Intracellular NO level in fungal hyphae 24 h and 48 h after MS-DBD plasma treatment for 120 s and 300 s. DIC: differential interference contrast, DAF-FM DA: fluorescence, Merge: combined image of DIC and fluorescence. (**b** and **c**) Relative FPase activity, protein concentration, and specific FPase activity in media 24 and 48 h after fungal hyphae were treated with MS-DBD plasma, followed by the addition of avicel supplemented with cPTIO (10 mM) **b** or treated with SNP (0.1 mM) **c**. **d** Level of mRNA of *nit-10*,* nit-3*,* nit-6*,* nit-2*, and *nit-4* in fungal hyphae 4 h after MS-DBD plasma treatment. Each value is the mean of six or nine replicate measurements (three independent biological replicates and two-three technical replicates): * *p* < 0.05, ** *p* < 0.01, *** *p* < 0.001 as determined by Student’s *t*-test

Levels of intracellular NO stained with a fluorescent dye (intensity of the green fluorescence) increased after MS-DBD plasma treatment (Fig. 5a and Supplementary Figure S8). We found that the relative FPase activity, total protein concentration, and specific FPase activity in the media significantly decreased after cPTIO (an NO scavenger) was added to both untreated and treated plasma samples (Fig. 5b and Supplementary Table S4a). When fungal hyphae (no plasma treated) were treated with SNP (exogenous NO donor), the relative FPase activity and total protein concentration in the media significantly increased without a significant change in relative specific FPase activity, indicating that extracellular NO accelerates the secretion of all proteins, not specifically cellulases (Fig. 5c and Supplementary Table S4b).

Since intracellular NO can be generated during nitrate reduction in cells, we also examined the expression of enzymes involved in nitrate assimilation. NO_3_^−^ can enter cells through nitrate transporters on the cell membrane and is gradually reduced to NO_2_^−^ and then to NO by nitrate reductase (NR) and nitrite reductase (NiR) (Bender and Schwarz 2018; Marcos et al. 2016). The mRNA expression levels of the nitrate transporter (*nit-10*), nitrate reductase (*nit-3*), nitrite reductase (*nit-6*), and positive regulators of *nit-3* (*nit-2* and *nit-4*) did not change significantly after plasma treatment (Fig. 5d).

**Fig. 6:**
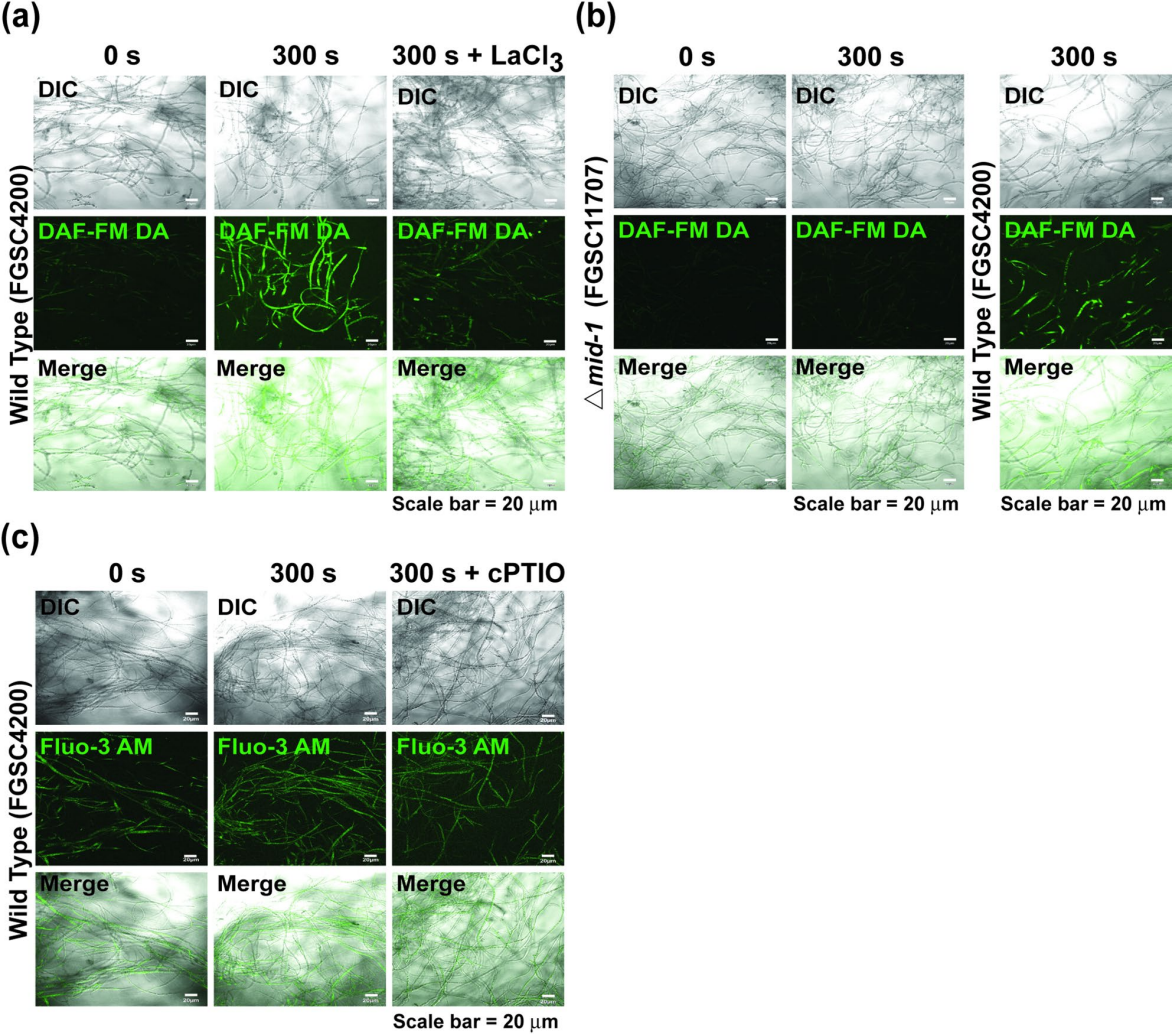
Relationship between intracellular NO and intracellular Ca^2+^. **a** Intracellular NO level in hyphae after 24 h of 5 mM LaCl_3_ treatment. **b** Intracellular NO levels in hyphae of *mid-1* mutant (FGSC11707) and wild type after 24 h of MS-DBD plasma treatment. **c** Intracellular Ca^2+^ levels in hyphae after 48 h of 10 mM cPTIO treatment. LaCl_3_ and cPTIO were added immediately after DBD plasma treatment with avicel. DIC: differential interference contrast, DAF-FM DA and Fluo-3 AM: fluorescence, Merge: combined image of DIC and fluorescence

Our results demonstrated an increase in the intracellular levels of Ca^2+^ and NO after plasma treatment. We explored the relationship between the Ca^2+^ and NO levels in fungal cells after plasma treatment. The addition of LaCl_3_ significantly reduced intracellular NO levels under MS*-*DBD plasma treatment (Fig. 6a and Supplementary Figure S9a). The intracellular NO levels in the *mid-1* mutant strain (FGSC11707) were significantly lower than those in the wild-type strain (FGSC 4200) after plasma treatment under the same conditions (Fig. 6b and Supplementary Figure S9b). In contrast, the addition of cPTIO did not dramatically affect intracellular Ca^2+^ levels in MS-DBD plasma treated hyphae (Fig. 6c and Supplementary Figure S10).

***“Plasma dosage” effect on the efficiency of fungal cellulase production***.

**Fig. 7:**
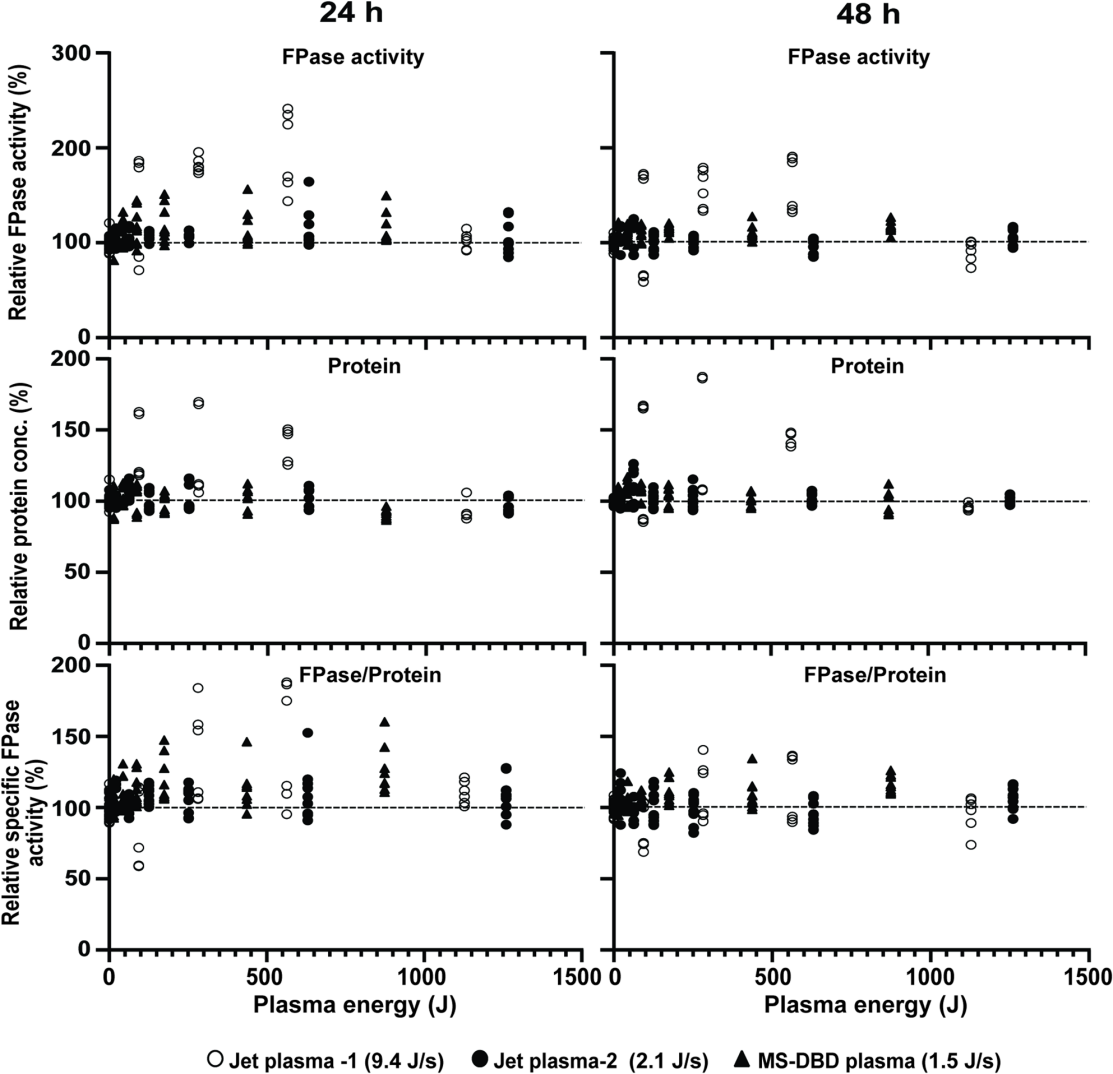
Relationship between plasma energy and cellulase production efficiency. Relative filter paper enzyme (FPase) activity, relative total protein concentration, and relative specific FPase activity in avicel media (Avicel was added before plasma treatment) assessed according to the value of plasma energy after treatment with different types of plasma (1.5 J/s MS-DBD plasma, 9.4 J/s Jet plasma-1, 2.1 J/s Jet plasma-2). Plasma energy was calculated as follows: plasma energy (J) = plasma power (J/s) × treatment time (s). Relative percentage values of plasma-treated samples (all replicates) compared to those of non-plasma-treated samples presented in graphs. All experiments were performed with at least three independent biological replicates, each with two-three technical replicates

To clarify the relationship between “plasma dose (plasma type, electric power, and treatment time)” and cellulase production efficiency, we used three types of plasma with different powers (1.5 J/s MS*-*DBD plasma, 9.4 J/s Jet plasma-1, and 2.1 J/s Jet plasma-2) to treat fungal hyphae in media containing avicel (inducer). Although different types of plasma with different powers were used, we defined the “plasma dose” as the plasma energy (electric power of plasma × treatment time) and compared the enzyme production efficiency according to plasma energy to determine the generalized dosage effect. When all replicate data points corresponding to the plasma energy were placed on the graph, there was a range of plasma energies resulting in the enhancement of FPase activity, protein concentration, and specific FPase activity (Fig. 7 and Supplementary Table S5). The activity of cellulases in the media increased under upto approximately 500–600 J of plasma energy (Fig. 7 and Supplementary Table S5).

Our results demonstrated that all types of plasma (Jet and MS-DBD) promoted the production of cellulases by *N. crassa* in the avicel medium (Fig. 7 and Supplementary Table S5). However, under the same plasma energy, jet plasma with high power (9.4 W) showed the highest efficiency in enzyme production (~ 200%) when treated for a short time (within 60 s) compared to jet plasma with low power (2.1 W) and MS-DBD plasma (Fig. 7 and Supplementary Table S5). In addition, the efficiency of cellulase production decreased with longer incubation times. The relative increase in percentage was reduced after 48 h of incubation compared to that after 24 h of incubation (Fig. 7). Enzyme activity was also elevated in glucose media in several plasma treatments (Supplementary Table S5). However, the relative FPase activity and specific enzyme activity increased in a plasma energy-dependent manner only under induction conditions.

### Potential of plasma as a tool for enhancing the efficiency of industrial-scale production of cellulases

**Fig. 8:**
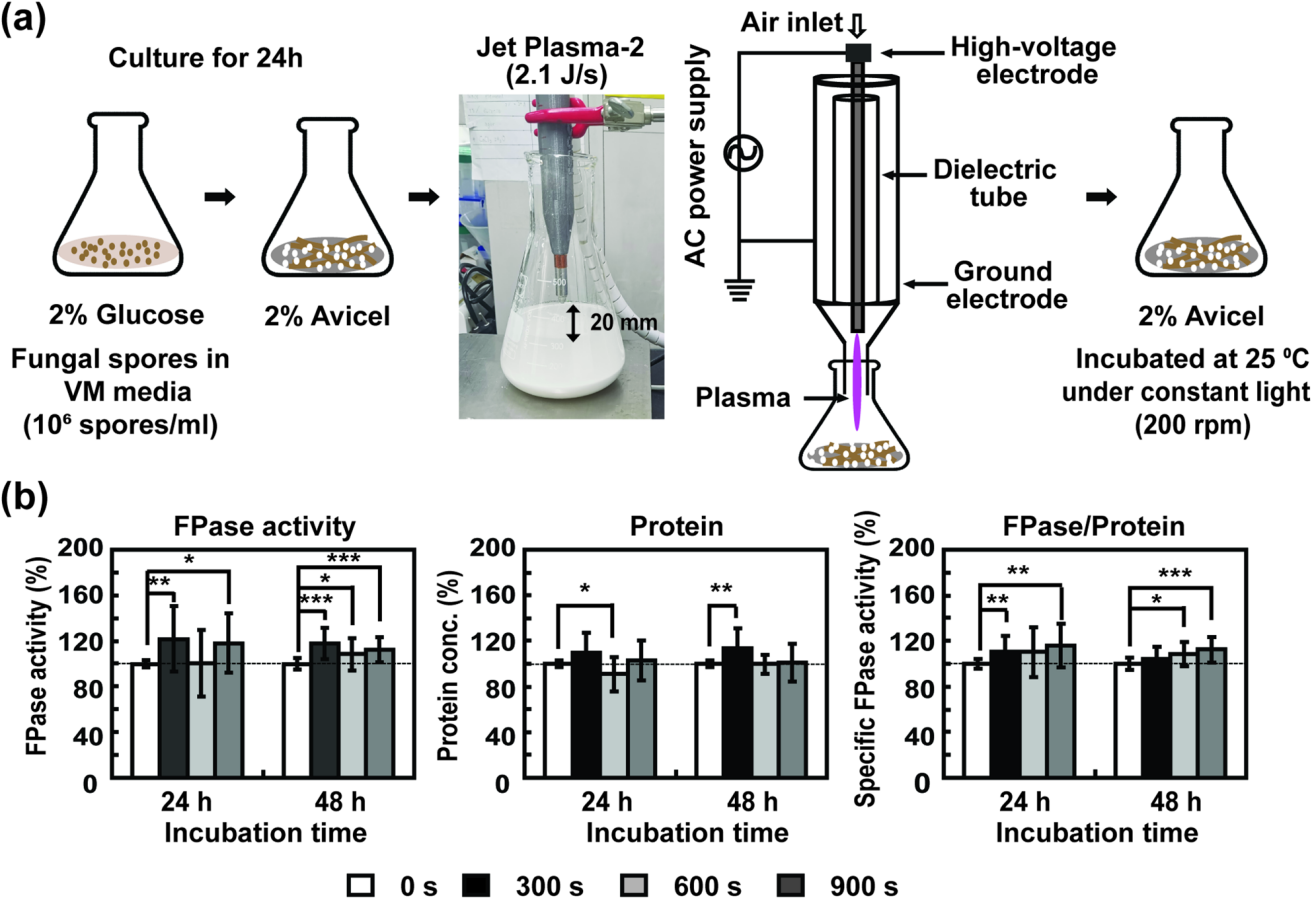
Effects of plasma on large-scale cellulase production. **a** Experimental scheme for 2.1 J/s Jet plasma-2 treatment of *N. crassa* hyphae in VM media (Avicel was added before plasma treatment). **b** Under large-scale culture conditions (300 mL), relative FPase activity, relative total protein concentration, and relative specific FPase activity in avicel medium after 2.1 J/s Jet plasma-2 treatment. The relative percentage was calculated compared to the value of the non-treated group. Each value is the mean of six or nine replicate measurements (three independent biological replicates and two-three technical replicates): * *p* < 0.05, ** *p* < 0.01, *** *p* < 0.001 as determined by Student’s *t*-test

The effect of a large-scale (300 mL; 10-fold increase in fungal culture volume) treatment was also analyzed to examine the potential of plasma for industrial-scale applications. For this experiment, we used jet plasma with 2.1 J/s power because jet plasma was more effective than MS-DBD plasma, and the treatment area of MS-DBD plasma was limited (Fig. 8a). The total enzyme activity in the media (with avicel) was significantly elevated up to a maximum of 20%, compared with the untreated group, after 24 and 48 h of plasma treatment for 300–900 s (Fig. 8b and Supplementary Table S6). The total protein content in the media did not significantly change in most treatment groups, and the specific enzyme activity was significantly elevated after 24 and 48 h of plasma treatment for 300–900 s (Fig. 8b and Supplementary Table S6). No significant increase in enzyme activity was observed in media without avicel (only glucose) (Supplementary Table S6). This indicates that jet plasma can enhance fungal cellulase production on a large scale in the presence of avicel (an inducer).

## Discussion

This study is a deepened and subsequent work to our previous research, representing substantial progress in technical approaches and mechanistic understanding. In this study, we provided another experimental evidence that non-thermal atmospheric-pressure plasma enhances the extracellular production of cellulases by *N. crassa* using plane-type MS-DBD plasma and established the relationship between plasma treatment dosage and enzyme activity. In terms of mechanism, we have clarified that the increase in intracellular Ca²⁺ levels is due to the activation of the high-affinity calcium ion influx system (HACS) on the cell membrane. Moreover, we have determined that Ca²⁺ acts upstream of NO in the plasma-induced signaling cascade.

Plane-type MS-DBD plasma was generated between microelectrodes embedded in a glass plate, resulting in the entire surface area of the plate (diameter 90 mm) being filled with plasma. In contrast, in jet plasma, plasma is generated in feeding gas nozzle. The plasma plume and sample cannot come into direct contact in treatment with plane-type MS-DBD plasma, whereas direct contact between the plasma plume and sample is possible in treatment with jet plasma. Our results indicate that jet plasma treatment is more effective at enhancing enzyme production than plane-type MS-DBD plasma treatment. After 2–5 min of treatment, a maximum increase of approximately 10% in cellulase activity in the media was observed with MS-DBD plasma in this study, whereas a 20% increase was observed with jet plasma (Yu et al. 2022). Interestingly, jet plasma treatment at higher power (9.4 W) for short duration was more effective in increasing fungal enzyme production than treatment at lower power (2.1 W). Our qPCR analysis suggests that the increased enzyme production results from an elevated expression of cellulase genes. Importantly, we applied plasma treatment to vegetative hyphae rather than spores, as this approach directly reflects industrial cellulase production systems where enzymes are secreted by actively growing mycelia in bioreactors.

Studies on plasma-mediated enhancement of microbial enzyme production are limited. Several studies have focused on plasma-mediated mutagenesis to generate microbial strains with high enzymatic activity (Li et al. 2025; Zhang et al. 2014). These studies used a very high dose of plasma, which caused the death of over 90% of the fungi. However, in our study, a low dose of plasma (most microorganisms were alive) was applied to the microorganisms, and this treatment condition might not be enough to cause microbial mutations. Very few studies have reported results similar to ours, in that plasma can promote enzyme expression and secretion in non-mutated microbial strains (Kabarkouhi et al. 2024; Farasat et al. 2018). Genetically engineered strains harboring recombinant enzyme genes have frequently been developed for industrial-scale production (Dadwal et al. 2020). However, the limited efficiency of protein expression and secretion can be a bottleneck for the application of engineered strains in the production industry. The results from our and other studies suggest that plasma can be a potential and safe tool for improving the efficiency of extracellular enzyme production without causing mutations. In several studies, catalytic activity was improved through non-mutagenic applications of plasma such as plasma pretreatment of substrates, enzymes, or a mixture of substrates and enzymes (Abolore et al. 2025; Chen et al. 2025; Dirks et al. 2025; Wapshott-Stehli et al., 2022; Yayci et al. 2020). In these studies, the improvement of catalytic activity through influence on enzyme structure or substrate susceptibility was the main target for analysis after plasma treatment.

Our results demonstrated that plasma-mediated enhancement of fungal enzyme production was observed in avicel media but not in glucose media. Avicel is a microcrystalline form of cellulose, and fungal cellulolytic enzyme production is induced by the addition of avicel to media (Coradetti et al. 2013). The degradation of cellulose by conidial-bound cellulases is initiated, producing disaccharide cellobiose, which enters the cells and further activates the transcription and expression of cellulase (Suto and Tomita 2001; Yan et al. 2021). The concentration of cellobiose is proportional to the induction effect, as these molecules are transported into fungal cells through transporters on the cell membrane and induce the expression of cellulase genes (Yan et al. 2021). In our study, it is possible that plasma accelerated the direct degradation of avicel in media, and cellobiose, the degradation product, was transported into the cell and induced cellulase production. However, plasma did not seem to be involved in the direct degradation of cellulose in media in our study because cellulose was added to the medium after plasma treatment (no contact with plasma). In addition, no significant change in the level of reducing sugars (no elevation of degradation) was observed when plasma was directly applied to the avicel medium for the time interval used in our study (Supplementary Figure S11a). Another possibility is that plasma treatment reduces the stability of the cellulose structure, making cellulose more easily and rapidly degraded by cellulase, ultimately increasing the concentration of cellobiose in the culture environment and accelerating the production of fungal cellulase.

Our cellular and molecular analyses provide several clues in terms of the mechanisms underlying the enhancement of cellulase production by plasma. The plasma-mediated elevation of cellulase production may have resulted from enhanced fungal growth after plasma treatment. This may be plausible because we observed a significant increase in fungal hyphal dry weight 48 h after plasma treatment for a time duration (120–300 s) showing the promotion of enzyme production (Supplementary Figure S11b). The increased hyphal dry weight observed under optimal plasma treatment conditions provides direct evidence that fungal viability was maintained, as significant cell death would have resulted in reduced biomass rather than the observed increase. A significant increase in enzyme production was observed after 24 h of incubation; however, fungal growth did not always show a significant increase during 24 h incubation (Supplementary Figure S11b). This indicates that, beyond fungal growth, other factors affected by plasma may lead to enhanced enzyme production. Additionally, our results also indicate that plasma treatment can influence cellulase production through indirect media-mediated effects. When fungi were cultured in plasma-treated medium, FPase activity and protein concentration were significantly increased (Fig. 2d and Supplementary Table S2). This suggests that plasma-induced changes in medium chemistry (increased levels of H₂O₂ and NOₓ, as shown in Fig. 2b) contribute to the enhancement of enzyme production. Multiple lines of evidence indicate that plasma also exerts direct effects on fungal cells. Immediate cellular responses observed after plasma treatment, including membrane potential depolarization (Supplementary Figure S3a) and chemical alterations in cell membrane composition (Fig. 3c; Table 2), suggest direct plasma-cell interactions. Furthermore, the transcriptional activation of cellulase genes (Fig. 1c) demonstrates that plasma directly triggers intracellular signaling pathways regulating cellulase gene expression. Taken together, our results support a dual-mechanism synergistic model in which plasma enhances cellulase production through both direct cellular effects and indirect medium-mediated effects.

Another possibility is that plasma may have changed the physical and chemical properties of the cell membrane, such as the membrane potential and chemical properties, which may have affected the opening of membrane transporters and channels, leading to ion and sugar influx. According to our data, the *N. crassa* cell membrane appeared to be depolarized after plasma treatment. In addition, Raman spectroscopy suggested chemical alterations in the lipids and proteins of the cell membrane and the glucans of the cell wall after plasma treatment. Depolarization of mammalian cell membranes can trigger the opening of Ca^2+^ channels or Ca^2+^ pumps (Catterall 2011; Csernoch et al. 2004). In addition, our study found that after plasma treatment, intracellular Ca^2+^ levels significantly increased, whereas the mRNA expression level of *PLC*, a signal that regulates the release of intracellular Ca^2+^ stores, did not change significantly. Therefore, we speculated that plasma treatment could trigger the opening of Ca^2+^ channels in the cell membrane by inducing membrane depolarization, leading to an influx of extracellular Ca^2+^. The Ca^2+^ influx system in fungal cell membranes is primarily composed of *Cch1p*, *Mid1p*, and *Ecm*7 (Lange and Peiter 2019). When the *mid-1* mutant strain (Ca^2+^ channel deletion) was treated with plasma, its ability to produce cellulase did not increase. Similarly, when cell membrane Ca^2+^ channel blockers (LaCl_3_) were added, the promoting effect of plasma on fungal cellulase production disappeared. This suggests that plasma promotes the production of fungal cellulases by increasing the influx of Ca^2+^ through channels on the cell membrane. Previous research has demonstrated that Ca²⁺ enhances cellulase production via the calmodulin/Crz1 signaling pathway (Li et al. 2022) and has characterized the regulatory relationship between calcium ions and cellulase transcription factor (Clr-2) (Li et al. 2022; Randhawa et al. 2024). Therefore, our study focused on elucidating the upstream signaling events triggered by plasma treatment. Specifically, we identified that plasma activates the Mid-1-mediated high-affinity Ca²⁺ influx system on the cell membrane, which subsequently regulates intracellular NO levels and ultimately modulates cellulase production.

The increase in intracellular NO levels after the plasma treatment observed in our study may also have played a role in promoting cellulase production. NO is an important regulatory molecule in the process of plasma-induced cellulase production. Intracellular NO participates in the regulatory network of cellulolytic enzyme production by regulating cAMP and MAPK (Yu et al. 2023). Several studies have shown that intracellular NO levels are elevated by nitrite-dependent NO production, nitric oxide synthase (NOS)-dependent NO production, and free diffusion of extracellular NO (Andrabi et al. 2023; Liu et al. 2002; Neill et al. 2003). In nitrite-dependent NO production, NO_3_^−^ in the media is taken up by the cell through the transporter and is assimilated into NH_3_, and NO can be produced as an intermediate during this process. In our study, the mRNA expression levels of genes involved in nitrite-dependent NO production, such as nitrate transporter (*nit-10*), nitrate reductase (*nit-3*), and nitrite reductase (*nit-6*), did not change significantly after plasma treatment. This suggests that plasma may increase intracellular NO level through nitric oxide synthase (NOS)-dependent NO production or free diffusion of extracellular NO. However, these conjectures require further verification, which will be the focus of future research. Several studies have reported the crosstalk between Ca^2+^ and NO (Besson-Bard et al. 2008; Jeandroz et al. 2013). In *Ganoderma lucidum*, the interaction between Ca^2+^ and NO regulates the gibberellic acid biosynthesis of ganoderma acid under heat stress (Liu et al. 2018). Our results suggest that plasma accelerates Ca^2+^ influx into fungal cells, leading to NO generation. While cellulase transcription is regulated by multiple transcription factors, the regulatory relationship between Ca²⁺ and both Clr-2 and Crz-1 has been well established, and Clr-1 is also considered a key cellulase transcription factor (Li et al. 2022; Randhawa et al. 2024). Thus, the downstream transcriptional machinery involving Clr-2 and Crz-1, which serve as master regulators of cellulase gene expression, likely constitutes the ultimate effector target of the Ca²⁺-NO signaling cascade revealed. Our results demonstrated significant upregulation of four major cellulase genes (*cbh-1*, *gh6-2*, *gh5-1*, and *gh3-4*) following plasma treatment, it is reasonable to hypothesize that Crz-1 and Clr-2 activity is enhanced through the plasma-triggered Ca²⁺-NO signaling pathway. Investigating the expression levels, phosphorylation status, and nuclear translocation of Crz-1 and Clr-2 in response to plasma treatment represents a critical direction for our future research to establish the complete signaling network.

Our data demonstrates that there may be a range of plasma dose activating enzyme production. However, a standard unit for the plasma dose has not been established, but the plasma energy (electric power × treatment time) applied to the sample has been frequently used as the plasma dose. Our results indicate that the upper limit of plasma energy for the promotion of enzyme production is 500–600 J, regardless of the plasma source type and feeding gases. With the same plasma energy, treatment with high-electric-power jet plasma for a short time appears to be more efficient for enzyme production. This indicates that the plasma treatment method (plasma type and a combination of high-power and short-term treatment) is also critical for improving the efficiency of enzyme production. Our data also suggests that jet plasma can be used to enhance the large-scale production of cellulases. Although a maximum 20% enhancement in enzyme production was obtained, the electric power of the plasma source and the treatment time should be optimized.

Compared to conventional enhancement methods for industrial enzyme production, plasma treatment offers distinct advantages in terms of cost-effectiveness, safety, and scalability. Traditional approaches such as chemical mutagenesis or adaptive evolution require extensive screening processes and prolonged strain development timelines (often several months to years), with potential concerns regarding genetic stability and regulatory approval for industrial deployment (Lee and Kim 2015). Genetic engineering strategies, while effective, face similar regulatory hurdles and public acceptance issues regarding genetically modified organisms (GMOs) (Rozas et al. 2022). Chemical induction methods, though widely used, incur ongoing costs for inducer compounds and necessitate subsequent removal of chemical residues from products (Lambert and Meers 1983). In comparison, plasma treatment represents a physical, rapid and non-mutagenic intervention.

Although the 6–10% increase observed with MS-DBD plasma may appear modest, from an industrial perspective, even a modest percentage improvement can have a significant economic impact. Plasma treatment equipment is relatively simple to construct, uses air as the feeding gas (low operational cost), and can be easily integrated into existing bioreactor systems without major infrastructure changes (Jiang et al. 2014). Moreover, it has a cumulative effect on large-scale cellulase production. In the industrial cellulase production valued at billions of dollars annually, a 6–10% yield improvement can translate into a significant increase in profitability (Ranjan et al. 2023). Any yield improvement achieved without additional substrate costs is valuable. Most importantly, plasma represents a novel, genetically safe, and scalable approach that complements rather than replaces existing production strategies. Moreover, our results demonstrate that further optimization of plasma parameters (particularly using high-power jet plasma achieving up to 96.30 ± 42.00% enhancement within 60 s, as shown in Fig. 7) indicates substantial potential for greater improvements. These findings provide valuable foundational knowledge for developing plasma-based enhancement strategies applicable to industrial-scale fungal enzyme production systems.

## Conclusions

In this study, we provide experimental evidence demonstrating the plasma (Jet and MS-DBD) -mediated enhancement of fungal enzyme production. Further investigation is required to determine whether plasma affects intracellular expression or extracellular secretion of enzymes. The promoting effect of plasma (Jet and MS-DBD) on enzyme production was observed when the plasma energy was below 600 J, indicating that plasma can exert a double-edged effect, ranging from activation to inactivation. However, the combination of plasma source type, electric power, and treatment duration can generate different outcomes, even under the same plasma energy. Plasma (MS-DBD) treatment can cause cell membrane depolarization, affect molecular transport, and elevate intracellular levels of secondary signaling molecules such as Ca^2+^ and NO. These results provide insight into the mechanisms of plasma-mediated enhancement of enzyme production. This non-mutagenic approach may be genetically and environmentally safe for large-scale fungal enzyme production and can be applied to bioreactors. However, establishing optimal conditions for plasma treatment is essential for the future application prospects of plasma.

## Supplementary Information

Below is the link to the electronic supplementary material.

Supplementary Material 1

## Data Availability

The datasets used and/or analysed during the current study are available from the corresponding author on reasonable request.
