# Supplementary material for: Non-thermal atmospheric-pressure plasma promotes cellulase production in Neurospora crassa
Source: Bioresour Bioprocess. 2026 Jan 27;13(1):13. doi: 10.1186/s40643-025-01006-z (PMC12835481; doi:10.1186/s40643-025-01006-z)
Supplement: Supplementary file 1 — Supplementary Material 1 [file 40643_2025_1006_MOESM1_ESM.docx]

**Supplementary Information**

**Non-thermal atmospheric-pressure plasma promotes cellulase production in *Neurospora crassa***

Nan-Nan Yu^a,b^, Wirinthip Ketya^a^, Kirubel Amsalu^c^, Jun-Sup Lim^a^, Hu-Nan Sun^b^, Eun-Ha Choi^a,c^, Gyungsoon Park^a,c,*^

^a^ Plasma Bioscience Research Center, Department of Plasma-Bio Display, Kwangwoon University, Seoul 01897, Korea

^b^ College of Life Science and Technology, Heilongjiang Bayi Agricultural University, Daqing, Heilongjiang 163319, P.R. China

^c^ Department of Electrical and Biological Physics, Kwangwoon University, Seoul 01897, Korea

^*^ Corresponding Author

Department of Electrical and Biological Physics, Kwangwoon University

20 Kwangwoon-ro, Nowon-gu, Seoul 01897, Korea

Phone: +82-2-940-8324

Fax: +82-2-940-5664

Email: gyungp@kw.ac.kr

**Supplementary Table S1.** Filter paper enzyme (FPase) activity, total protein concentration, specific FPase activity in glucose or avicel media after MS-DBD plasma treatment

| **Glucose** | | | | |
| --- | --- | --- | --- | --- |
| Incubation time | Plasma treatment time (second) | FPase activity (IU^a^/mL) | Protein concentration (μg/mL) | Specific FPase activity (IU/mg) |
| 24 h | 0 | 0.038 ± 0.005^b^ | 34.755 ± 9.461 | 1.156 ± 0.229 |
|  | 10 | 0.039 ± 0.003 | 36.034 ± 11.078 | 1.152 ± 0.302 |
|  | 30 | 0.038 ± 0.004 | 33.213 ± 13.886 | 1.271 ± 0.390 |
|  | 60 | 0.039 ± 0.003 | 28.970 ± 15.981 | 1.729 ± 0.798 |
|  | 120 | 0.038 ± 0.004 | 31.248 ± 11.040 | 1.337 ± 0.427 |
|  | 300 | 0.038 ± 0.005 | 34.461 ± 11.281 | 1.234 ± 0.457 |
|  | 600 | 0.039 ± 0.002 | 25.493 ± 16.813 | 2.150 ± 1.131* |
| 48 h | 0 | 0.038 ± 0.003 | 72.773 ± 41.663 | 0.672 ± 0.331 |
|  | 10 | 0.038 ± 0.003 | 69.386 ± 33.459 | 0.669 ± 0.298 |
|  | 30 | 0.038 ± 0.003 | 69.365 ± 36.660 | 0.714 ± 0.406 |
|  | 60 | 0.040 ± 0.005 | 84.280 ± 55.584 | 0.639 ± 0.311 |
|  | 120 | 0.038 ± 0.003 | 69.146 ± 38.050 | 0.743 ± 0.429 |
|  | 300 | 0.038 ± 0.006 | 72.827 ± 35.851 | 0.609 ± 0.214 |
|  | 600 | 0.040 ± 0.004 | 64.298 ± 34.774 | 0.759 ± 0.306 |
| **Avicel** | | | | |
| Incubation time | Plasma treatment time (second) | FPase activity (IU/mL) | Protein concentration (μg/mL) | Specific FPase activity (IU/mg) |
| 24 h | 0 | 0.248 ± 0.015 | 158.159 ± 21.301 | 1.587 ± 0.197 |
|  | 10 | 0.261 ± 0.014* | 178.577 ± 7.463** | 1.466 ± 0.112 |
|  | 30 | 0.253 ± 0.022 | 170.220 ± 6.974 | 1.491 ± 0.170 |
|  | 60 | 0.246 ± 0.023 | 159.357 ± 5.717 | 1.544 ± 0.153 |
|  | 120 | 0.267 ± 0.021** | 157.607 ± 20.318 | 0.172 ± 0.257*** |
|  | 300 | 0.273 ± 0.033* | 159.176 ± 17.588 | 0.174 ± 0.303** |
|  | 600 | 0.263 ± 0.035 | 163.535 ± 4.973 | 1.607 ± 0.194 |
| 48 h | 0 | 0.289 ± 0.051 | 210.062 ± 7.648 | 1.381 ± 0.274 |
|  | 10 | 0.292 ± 0.026 | 224.191 ± 11.459** | 1.301 ± 0.072 |
|  | 30 | 0.272 ± 0.042 | 212.648 ± 8.314 | 1.272 ± 0.152 |
|  | 60 | 0.275 ± 0.024 | 201.287 ± 9.087* | 1.182 ± 0.290 |
|  | 120 | 0.298 ± 0.052* | 209.253 ± 12.403 | 1.424 ± 0.233 |
|  | 300 | 0.299 ± 0.046* | 215.462 ± 9.785 | 1.384 ± 0.165 |
|  | 600 | 0.278 ± 0.035 | 203.824 ± 10.599 | 1.359 ± 0.110 |

^a^ IU: one International Unit (IU) of enzyme activity was defined as the amount of enzyme that released 1 µM of reducing sugar per minute.

^b^Each value is the mean of 6 or 9 replicate measurements (three independent biological replicates and two-three technical replicates) : * *p* < 0.05, ** *p* < 0.01, *** *p* < 0.001 as determined by Student’s t-test

Supplementary Table S2. Filter paper enzyme (FPase) activity, total protein concentration, and specific FPase activity measured in media at 24 and 48 h after fungal hyphae were placed in MS-DBD plasma-treated or untreated VM media and then avicel was immediately added (final concentration 2%).

| **Avicel** | | | | |
| --- | --- | --- | --- | --- |
| Incubation time | Plasma treatment time (second) | FPase activity (IU^a^ /mL) | Protein concentration (μg/mL) | Specific FPase activity (IU/mg) |
| 24 h | 0 | 0.191 ± 0.016^b^ | 120.453 ± 7.558 | 1.595 ± 0.242 |
|  | 120 | 0.218 ± 0.011 | 130.065 ± 2.268 | 1.674 ± 0.080 |
|  | 300 | 0.240 ± 0.001** | 131.267 ± 1.876 | 1.828 ± 0.029 |
| 48 h | 0 | 0.254 ± 0.001 | 181.430 ± 1.376 | 1.402 ± 0.013 |
|  | 120 | 0.307 ± 0.009*** | 194.647 ± 3.122** | 1.575 ± 0.063** |
|  | 300 | 0.283 ± 0.011* | 182.031 ± 2.384 | 1.552 ± 0.050** |

^a^ IU: one International Unit (IU) of enzyme activity was defined as the amount of enzyme that released 1 µM of reducing sugar per minute.

^b^ Each value is the mean of three biological replicate measurements. * *p* < 0.05, ** *p* < 0.01, *** *p* < 0.001 as determined by Student’s t-test.

**Supplementary Table S3**. Effects of inhibition or deletion of Ca^2+^ channel on cellulases production. **(a)** FPase activity, total protein concentration, and specific FPase activity in media. Wild type fungal hyphae in VM media were treated with MS-DBD plasma, immediately followed by the addition of avicel and LaC1_3_ (Ca^2+^ channel inhibitor). **(b)** FPase activity, total protein concentration, and specific FPase activity in media. Wild type and deletion mutant of Ca^2+^ channel (*Δmid-1*) were treated with MS-DBD plasma, immediately followed by the addition of avicel.

| Incubation time | Plasma treatment time (second) | Addition of LaCl_3_ | FPase activity (IU^a^/mL) | Protein concentration (μg/mL) | Specific FPase activity (IU/mg) |
| --- | --- | --- | --- | --- | --- |
| 24 h | 0 | **˗** | 0.227 ± 0.032^b^ | 137.102 ± 49.759 | 1.858 ± 0.653 |
|  | 300 | **˗** | 0.246 ± 0.029 | 151.427 ± 40.313 | 1.734 ± 0.486 |
|  | 0 | **+** | 0.102 ± 0.044 | 70.507 ± 39.369 | 1.729 ± 0.596 |
|  | 300 | **+** | 0.111 ± 0.058*** | 70.757 ± 43.008*** | 1.871 ± 0.585 |
| 48 h | 0 | **˗** | 0.271 ± 0.029 | 202.368 ± 54.692 | 1.407 ± 0.303 |
|  | 300 | **˗** | 0.265 ± 0.019 | 200.513 ± 40.728 | 1.367 ± 0.280 |
|  | 0 | **+** | 0.151 ± 0.013 | 111.853 ± 40.218 | 1.539 ± 0.603 |
|  | 300 | **+** | 0.143 ± 0.027*** | 112.907 ± 42.294*** | 1.423 ± 0.552 |

| Strain | Incubation time | Plasma treatment time (second) | FPase activity (IU/mL) | Protein concentration (μg/mL) | Specific FPase activity (IU/mg) |
| --- | --- | --- | --- | --- | --- |
| Wild type | 24 h | 0 | 0.227 ± 0.032 | 137.102 ± 49.759 | 1.858 ± 0.653 |
|  |  | 300 | 0.246 ± 0.029 | 151.427 ± 40.313 | 1.734 ± 0.486 |
|  | 48 h | 0 | 0.271 ± 0.029 | 202.368 ± 54.692 | 1.407 ± 0.303 |
|  |  | 300 | 0.265 ± 0.019 | 200.513 ± 40.728 | 1.367 ± 0.280 |
| *Δmid-1* | 24 h | 0 | 0.070 ± 0.013 | 48.667 ± 18.249 | 1.595 ± 0.469 |
|  |  | 300 | 0.057 ± 0.011* | 46.218 ± 17.304 | 1.353 ± 0.388 |
|  | 48 h | 0 | 0.253 ± 0.077 | 146.469 ± 60.805 | 1.826 ± 0.329 |
|  |  | 300 | 0.243 ± 0.067 | 138.423 ± 61.162 | 1.910 ± 0.511 |

^a^ IU: one International Unit (IU) of enzyme activity was defined as the amount of enzyme that released 1 µM of reducing sugar per minute.

^b^Each value is the average of 9 replicate measurements (three independent biological replicates and three technical replicates) : * *p* < 0.05, ** *p* < 0.01, *** *p* < 0.001 as determined by Student’s t-test.

**Supplementary Table S4**. Filter paper enzyme (FPase) activity, protein concentration, and specific FPase activity measured in media after MS-DBD plasma treatment, immediately followed by addition of avicel and cPTIO **(a)** or after SNP treatment **(b)**.

(a)

| Incubation time | Plasma treatment (second) | Addition of cPTIO | FPase activity (IU^a^/mL) | Protein concentration (μg/mL) | Specific FPase activity (IU/mg) |
| --- | --- | --- | --- | --- | --- |
| 24 h | 0 | **˗** | 0.209 ± 0.007^b^ | 174.194 ± 22.709 | 1.219 ± 0.183 |
|  | 300 | **˗** | 0.179 ± 0.023 | 169.623 ± 12.538 | 1.062 ± 0.155 |
|  | 0 | **+** | 0.028 ± 0.002 | 86.203 ± 14.566 | 0.332 ± 0.070 |
|  | 300 | **+** | 0.026 ± 0.003*** | 93.361 ± 27.543*** | 0.304 ± 0.095*** |
| 48 h | 0 | **˗** | 0.251 ± 0.041 | 243.040 ± 8.728 | 1.031 ± 0.135 |
|  | 300 | **˗** | 0.236 ± 0.033 | 239.031 ± 2.783 | 0.987 ± 0.134 |
|  | 0 | **+** | 0.070 ± 0.035 | 135.736 ± 7.933 | 0.509 ± 0.235 |
|  | 300 | **+** | 0.044 ± 0.006*** | 134.661 ± 23.194*** | 0.343 ± 0.097*** |

(b)

| Incubation time | SNP treatment (mM) | FPase activity (IU/mL) | Protein concentration (μg/mL) | Specific FPase activity (IU/mg) |
| --- | --- | --- | --- | --- |
| 24 h | 0 | 0.214 ± 0.017 | 133.893 ± 25.384 | 1.637 ± 0.249 |
|  | 0.1 | 0.259 ± 0.018*** | 158.896 ± 24.958 | 1.660 ± 0.207 |
|  | 0.5 | 0.226 ± 0.014 | 138.752 ± 10.912 | 1.636 ± 0.175 |
|  | 0.8 | 0.238 ± 0.028* | 136.933 ± 8.140 | 1.741 ± 0.205 |
| 48 h | 0 | 0.257 ± 0.031 | 170.055 ± 28.640 | 1.543 ± 0.281 |
|  | 0.1 | 0.298 ± 0.052 | 191.084 ± 36.186 | 1.573 ± 0.190 |
|  | 0.5 | 0.362 ± 0.030*** | 211.829 ± 34.904* | 1.747 ± 0.296 |
|  | 0.8 | 0.299 ± 0.011** | 179.223 ± 29.849 | 1.709 ± 0.284 |

^a^ IU: one International Unit (IU) of enzyme activity was defined as the amount of enzyme that released 1 µM of reducing sugar per minute.

^b^ Each value is the average of 6 or 9 replicate measurements (three independent biological replicates and two-three technical replicates) : * *p* < 0.05, ** *p* < 0.01, *** *p* < 0.001 as determined by Student’s t-test.

**Supplementary Table S5**. Filter paper enzyme (FPase) activity, total protein concentration and specific FPase activity in glucose or avicel media^a^ after 9.4 W plasma jet (a), 2.1 W plasma jet (b) or 1.5 W MS-DBD plasma (c) treatment.

(a)

| Media | Incubation time | 9.4 J/s Plasma jet treatment (second) | FPase activity (IU^b^/mL) | Protein concentration (μg/mL) | Specific FPase activity (IU/mg) |
| --- | --- | --- | --- | --- | --- |
| Glucose | 24 h | 0 | 0.049 ± 0.009^c^ | 8.517 ± 3.832 | 5.988 ± 2.749 |
|  |  | 10 | 0.057 ± 0.011 | 10.860 ± 1.641 | 5.303 ± 1.114 |
|  |  | 30 | 0.054 ± 0.004 | 12.313 ± 3.959 | 4.863 ± 1.962 |
|  |  | 60 | 0.053 ± 0.004 | 10.497 ± 3.690 | 6.409 ± 4.847 |
|  |  | 120 | 0.055 ± 0.009 | 8.193 ± 4.761 | 5.612 ± 2.588 |
|  |  | 300 | 0.041 ± 0.012 | 8.309 ± 5.100 | 3.501 ± 0.851 |
|  |  | 600 | 0.064 ± 0.034 | 9.704 ± 2.937 | 8.646 ± 6.455 |
|  | 48 h | 0 | 0.045 ± 0.006 | 28.076 ± 13.302 | 1.892 ± 0.692 |
|  |  | 10 | 0.047 ± 0.007 | 31.564 ± 19.139 | 2.290 ± 1.609 |
|  |  | 30 | 0.051 ± 0.007* | 35.802 ± 13.633 | 1.653 ± 0.806 |
|  |  | 60 | 0.050 ± 0.016 | 38.466 ± 13.357 | 1.377 ± 0.426 |
|  |  | 120 | 0.043 ±0.005 | 19.716 ± 1.398 | 2.214 ± 0.327 |
|  |  | 300 | 0.042 ± 0.005 | 26.573 ± 8.354 | 1.761 ± 0.635 |
|  |  | 600 | 0.041 ± 0.005 | 24.285 ± 5.875 | 1.752 ± 0.312 |
| Avicel | 24 h | 0 | 0.098 ± 0.047 | 68.927 ± 30.276 | 1.436 ± 0.306 |
|  |  | 10 | 0.138 ± 0.073 | 104.028 ± 3.474* | 1.343 ± 0.742 |
|  |  | 30 | 0.187 ± 0.019*** | 101.849 ± 4.216* | 1.833 ± 0.148** |
|  |  | 60 | 0.198 ± 0.026*** | 103.483 ± 11.587* | 1.918 ± 0.126** |
|  |  | 120 | 0.092 ± 0.061 | 56.538 ± 36.640 | 1.604 ± 0.092 |
|  |  | 300 | 0.087 ± 0.057 | 54.285 ± 29.678 | 1.508 ± 0.253 |
|  |  | 600 | 0.037 ± 0.004** | 26.880 ± 1.163** | 1.399 ± 0.199 |
|  | 48 h | 0 | 0.219 ± 0.083 | 123.416 ± 47.360 | 1.806 ± 0.214 |
|  |  | 10 | 0.243 ± 0.109 | 151.611 ± 21.722 | 1.541 ± 0.499 |
|  |  | 30 | 0.335 ± 0.024** | 178.308 ± 15.528* | 1.886 ± 0.112 |
|  |  | 60 | 0.350 ± 0.085** | 181.940 ± 32.652* | 1.903 ± 0.129 |
|  |  | 120 | 0.215 ± 0.131 | 113.732 ± 61.533 | 1.815 ± 0.225 |
|  |  | 300 | 0.210 ± 0.111 | 121.775 ± 57.761 | 1.689 ± 0.194 |
|  |  | 600 | 0.098 ± 0.023** | 68.506 ± 5.514* | 1.423 ±0.232** |

(b)

| Media | Incubation time | 2.1 J/s Plasma jet treatment (second) | FPase activity (IU/mL) | Protein concentration (μg/mL) | Specific FPase activity (IU/mg) |
| --- | --- | --- | --- | --- | --- |
| Glucose | 24 h | 0 | 0.045 ± 0.006 | 23.297 ± 4.416 | 1.979 ± 0.287 |
|  |  | 10 | 0.055 ± 0.006 | 28.817 ± 1.104 | 1.911 ± 0.149 |
|  |  | 30 | 0.055 ± 0.004 | 28.817 ± 2.921 | 1.898 ± 0.130 |
|  |  | 60 | 0.047 ± 0.005 | 29.186 ± 1.275 | 1.616 ± 0.106 |
|  |  | 120 | 0.044 ± 0.001 | 27.345 ± 2.298 | 1.628 ± 0.127 |
|  |  | 300 | 0.048 ± 0.006 | 31.762 ± 1.275* | 1.511 ± 0.235 |
|  |  | 600 | 0.046 ± 0.009 | 28.449 ± 0.637 | 1.621 ± 0.325 |
|  | 48 h | 0 | 0.045 ± 0.002 | 33.970 ± 0.637 | 1.331 ± 0.038 |
|  |  | 10 | 0.049 ± 0.008 | 37.650 ± 1.104** | 1.308 ± 0.214 |
|  |  | 30 | 0.043 ± 0.005 | 30.658 ± 0.637** | 1.387 ± 0.124 |
|  |  | 60 | 0.039 ± 0.004 | 35.442 ± 1.912 | 1.108 ± 0.071** |
|  |  | 120 | 0.040 ± 0.007 | 34.338 ± 0.000 | 1.163 ± 0.202 |
|  |  | 300 | 0.042 ± 0.005 | 34.706 ± 1.687 | 1.209 ± 0.107 |
|  |  | 600 | 0.040 ± 0.005 | 33.234 ± 1.104 | 1.206 ± 0.186 |
| Avicel | 24 h | 0 | 0.254 ± 0.014 | 160.618 ± 8.538 | 1.584 ± 0.095 |
|  |  | 10 | 0.261 ± 0.032 | 156.673 ± 5.055 | 1.663 ± 0.163 |
|  |  | 30 | 0.278 ± 0.015** | 172.390 ± 7.157** | 1.611 ± 0.079 |
|  |  | 60 | 0.273 ± 0.013** | 158.960 ± 12.331 | 1.725 ± 0.117* |
|  |  | 120 | 0.272 ± 0.010** | 163.880 ± 16.769 | 1.674 ± 0.171 |
|  |  | 300 | 0.290 ± 0.046* | 165.739 ± 8.349 | 1.754 ± 0.310 |
|  |  | 600 | 0.267 ± 0.039 | 155.028 ± 3.537 | 1.718 ± 0.225 |
|  | 48 h | 0 | 0.278 ± 0.044 | 197.106 ± 17.575 | 1.426 ± 0.296 |
|  |  | 10 | 0.294 ± 0.057 | 197.516 ± 19.350 | 1.501 ± 0.314 |
|  |  | 30 | 0.300 ± 0.038 | 216.817 ± 34.303 | 1.410 ± 0.241 |
|  |  | 60 | 0.280 ± 0.043 | 199.561 ± 15.267 | 1.415 ± 0.249 |
|  |  | 120 | 0.281 ± 0.051 | 201.443 ± 26.408 | 1.430 ± 0.386 |
|  |  | 300 | 0.268 ± 0.039 | 199.320 ± 17.317 | 1.354 ± 0.235 |
|  |  | 600 | 0.296 ± 0.054 | 196.725 ± 16.455 | 1.509 ± 0.285 |

(c)

| Media | Incubation time | 1.5 J/s MS-DBD Plasma treatment (second) | FPase activity (IU/mL) | Protein concentration (μg/mL) | Specific FPase activity (IU/mg) |
| --- | --- | --- | --- | --- | --- |
| Avicel | 24 h | 0 | 0.253 ± 0.030 | 171.066 ± 23.442 | 1.499 ± 0.233 |
|  |  | 10 | 0.262 ± 0.043 | 168.350 ± 20.803 | 1.562 ± 0.239 |
|  |  | 30 | 0.286 ± 0.012** | 175.038 ± 14.406 | 1.643 ± 0.166 |
|  |  | 60 | 0.291 ± 0.019** | 175.659 ± 19.306 | 1.668 ± 0.141 |
|  |  | 120 | 0.298 ± 0.023** | 170.663 ± 18.155 | 1.755 ± 0.130* |
|  |  | 300 | 0.288 ± 0.032* | 172.480 ± 23.459 | 1.681 ± 0.159 |
|  |  | 600 | 0.288 ± 0.027* | 155.962 ± 20.814 | 1.859 ± 0.168** |
|  | 48 h | 0 | 0.273 ± 0.036 | 222.593 ± 43.959 | 1.245 ± 0.134 |
|  |  | 10 | 0.299 ± 0.046 | 234.463 ± 42.387 | 1.288 ± 0.121 |
|  |  | 30 | 0.298 ± 0.037 | 235.490 ± 44.846 | 1.288 ± 0.150 |
|  |  | 60 | 0.302 ± 0.027 | 233.363 ± 39.395 | 1.315 ± 0.136 |
|  |  | 120 | 0.311 ± 0.041 | 229.350 ± 43.985 | 1.381 ± 0.181 |
|  |  | 300 | 0.303 ± 0.053 | 222.851 ± 37.061 | 1.366 ± 0.138 |
|  |  | 600 | 0.318 ± 0.035* | 223.476 ± 41.219 | 1.448 ± 0.169* |

^a^ Glucose or avicel was added before plasma treatment.

^b^ IU: one International Unit (IU) of enzyme activity was defined as the amount of enzyme that released 1 µM of reducing sugar per minute.

^c^Each value is the mean of 3 or 9 replicate measurements (three independent biological replicates and one-three technical replicates) : * *p* < 0.05, ** *p* < 0.01, *** *p* < 0.001 as determined by Student’s t-test.

**Supplementary Table S6**. Filter paper enzyme activity (FPase), total protein concentration and specific FPase activity in large-scale (300 mL) glucose or avicel media^a^ after 2.1 W plasma jet treatment.

| Media | Incubation time | Plasma treatment (second) | FPase activity (IU^b^/mL) | Protein concentration (μg/mL) | Specific FPase activity (IU/mg) |
| --- | --- | --- | --- | --- | --- |
| Glucose | 24 h | 0 | 0.065 ± 0.020^c^ | 24.737 ± 0.941 | 2.631 ± 0.745 |
|  |  | 300 | 0.032 ± 0.024 | 22.856 ± 0.000* | 1.403 ± 1.059 |
|  |  | 600 | 0.017 ± 0.013* | 22.856 ± 0.000* | 0.764 ± 0.574* |
|  |  | 900 | 0.026 ± 0.014* | 19.720 ± 1.437** | 1.343 ± 0.827 |
|  | 48 h | 0 | 0.058 ± 0.007 | 30.694 ± 1.437 | 1.895 ± 0.251 |
|  |  | 300 | 0.061 ± 0.007 | 28.185 ± 1.437 | 2.165 ± 0.234 |
|  |  | 600 | 0.050 ± 0.007 | 24.423 ± 1.086** | 2.052 ± 0.365 |
|  |  | 900 | 0.047 ± 0.010 | 24.423 ± 1.086** | 1.913 ± 0.311 |
| Avicel | 24 h | 0 | 0.106 ± 0.035 | 67.544 ± 8.436 | 1.542 ± 0.369 |
|  |  | 300 | 0.128 ± 0.046 | 73.501 ± 12.891 | 1.708 ± 0.451 |
|  |  | 600 | 0.100 ± 0.023 | 61.595 ± 12.794 | 1.660 ± 0.360 |
|  |  | 900 | 0.118 ± 0.020 | 68.518 ± 10.578 | 1.755 ± 0.379 |
|  | 48 h | 0 | 0.216 ± 0.026 | 111.345 ± 17.169 | 1.985 ± 0.365 |
|  |  | 300 | 0.256 ± 0.043** | 124.321 ± 5.280** | 2.067 ± 0.383 |
|  |  | 600 | 0.232 ± 0.023 | 110.916 ± 20.072 | 2.158 ± 0.468 |
|  |  | 900 | 0.242 ± 0.030* | 111.976 ± 21.602 | 2.222 ± 0.394 |

^a^ Glucose or avicel was added before plasma treatment.

^b^ IU: one International Unit (IU) of enzyme activity was defined as the amount of enzyme that released 1 µM of reducing sugar per minute.

^c^ Each value is the mean of 3 or 15 replicate measurements (at least three independent biological replicates and one-three technical replicates): * *p* < 0.05, ** *p* < 0.01, *** *p* < 0.001 as determined by Student’s t-test.


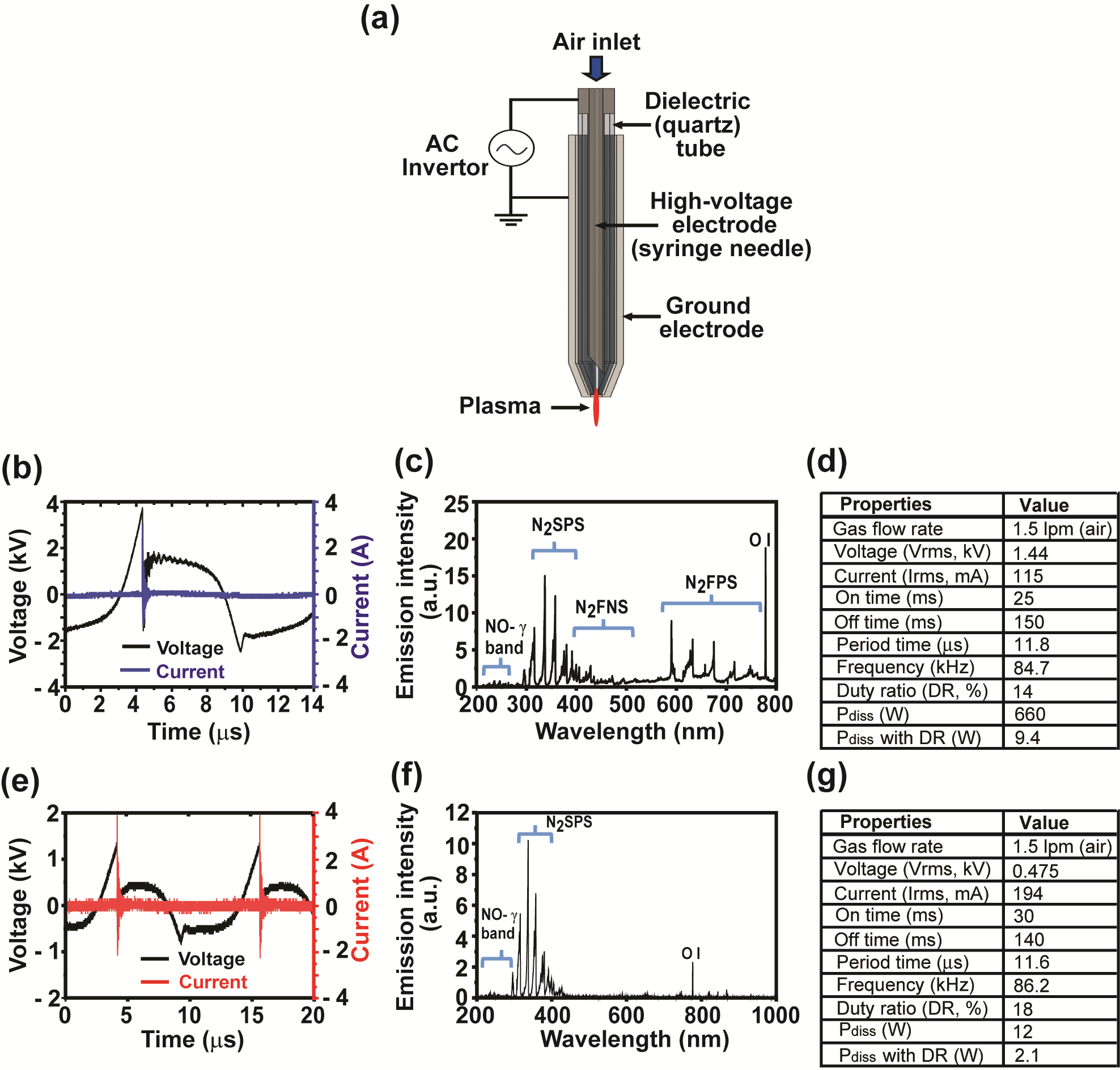


Supplementary Figure S1. Characterization of two plasma jets with different dissipated power, 9.4 W (Jet plasma-1) or 2.1 W (Jet plasma-2). (**a**) Schematic view of plasma jet, (**b-d**) voltage and current profile (**b**), OES spectra (**c**), and physical properties (**d**) of Jet plasma-1 with dissipated power 9.4 W. (**e-g**) voltage and current profile (**e**), OES spectra (**f**), and physical properties (**g**) of Jet plasma-2 with dissipated power 2.1 W.


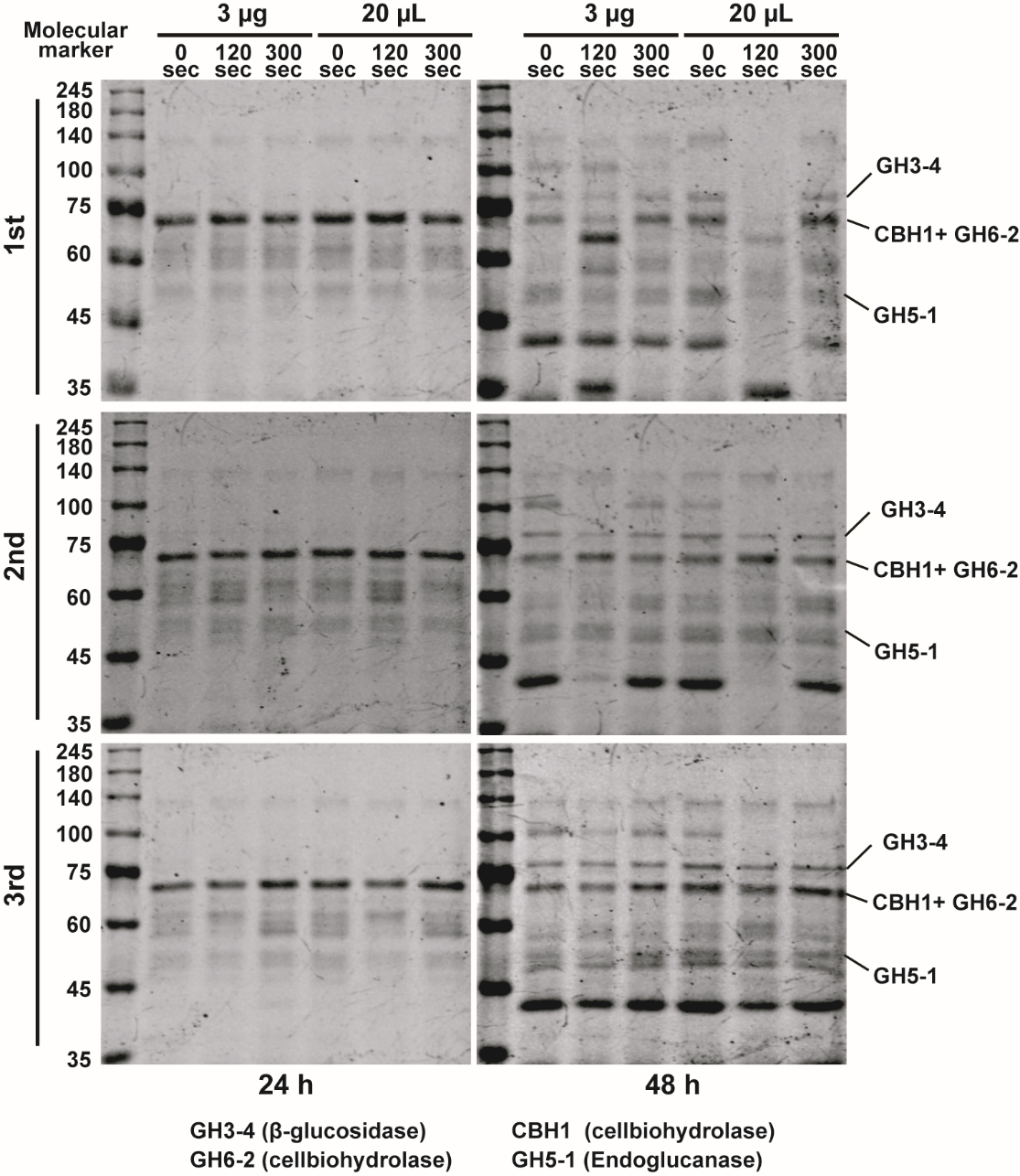


Supplementary Figure S2. Levels of four cellulolytic enzymes in avicel medium on SDS-PAGE gel. Fungal mycelia were removed by centrifugation, and culture supernatants were harvested at 24h and 48h after MS-DBD plasma treatment. The secreted proteins into media were resolved on SDS-PAGE gel, and gel pictures from three repeated experiments were shown. Bands corresponding to four cellulolytic enzymes are indicated.


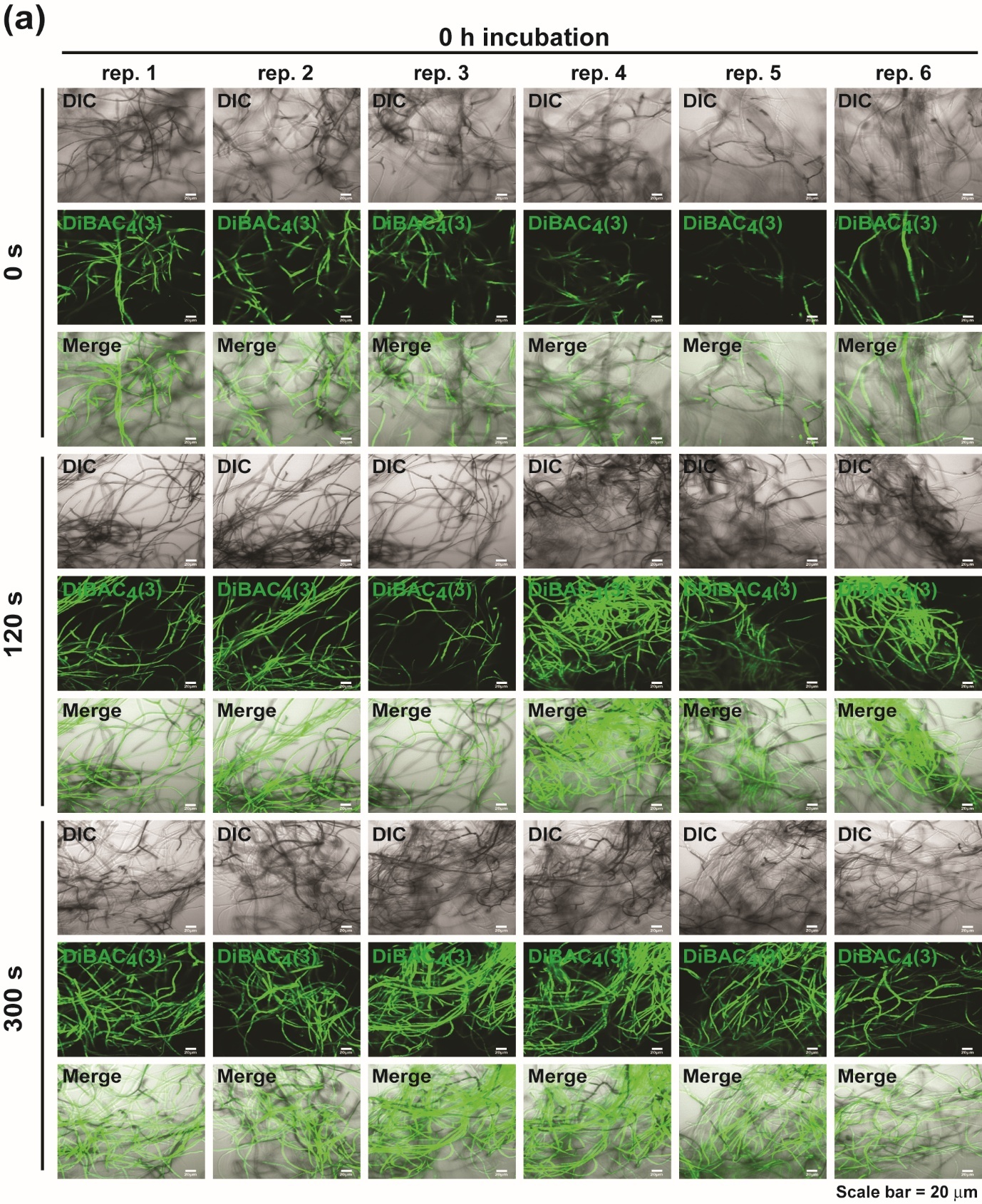


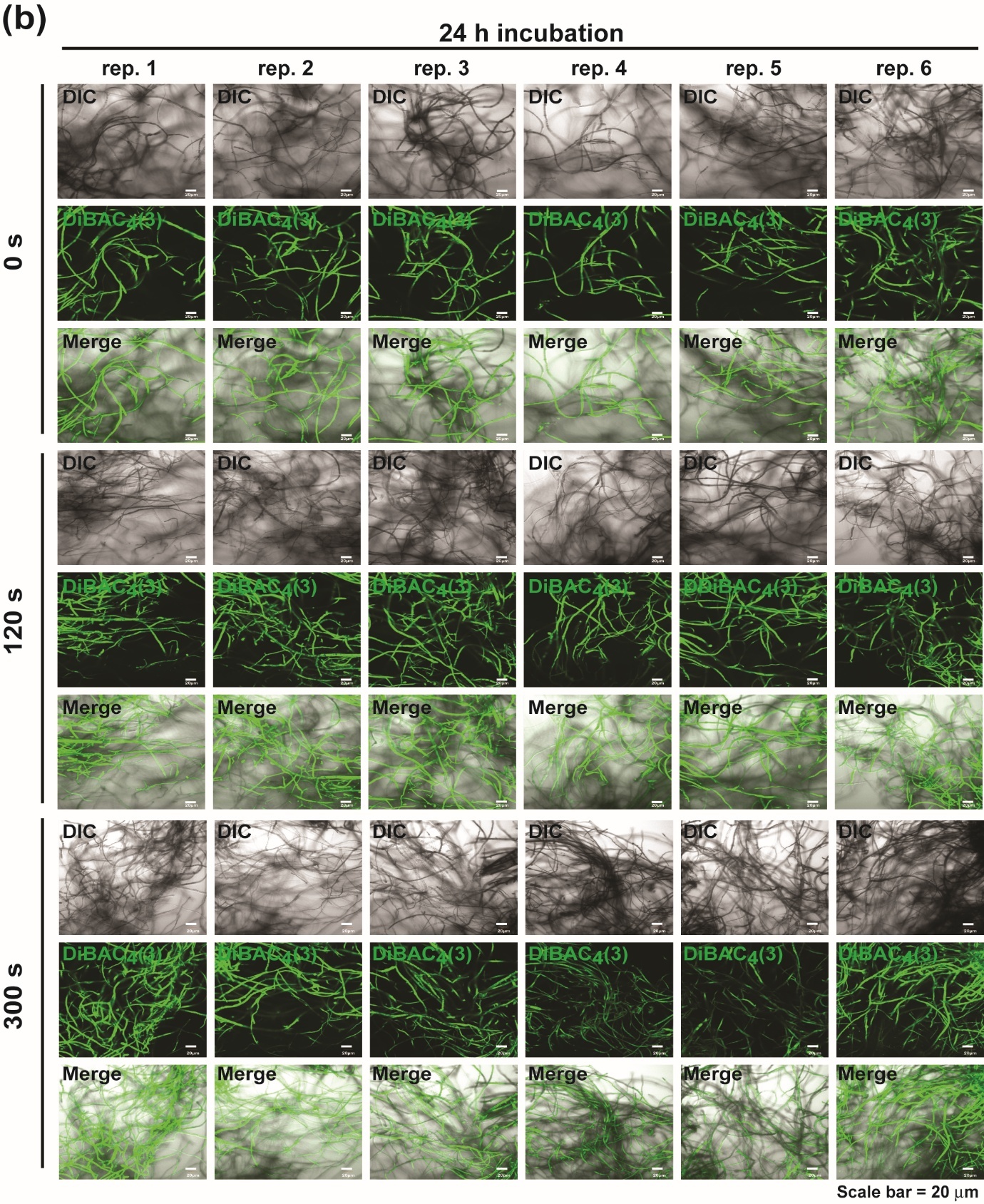


Supplementary Figure S3. Analysis of membrane potential in *N. crassa* hyphae after plasma treatment. Hyphae were stained with DiBAC_4_(3) (green fluorescence) after 0h (a) and 24h (b) of plasma treatment. Pictures of fungal hyphae were taken in 6 different areas. DIC; Differential Interference Contrast, DiBAC_4_(3); fluorescence, Merge; combined image of DIC and fluorescence.


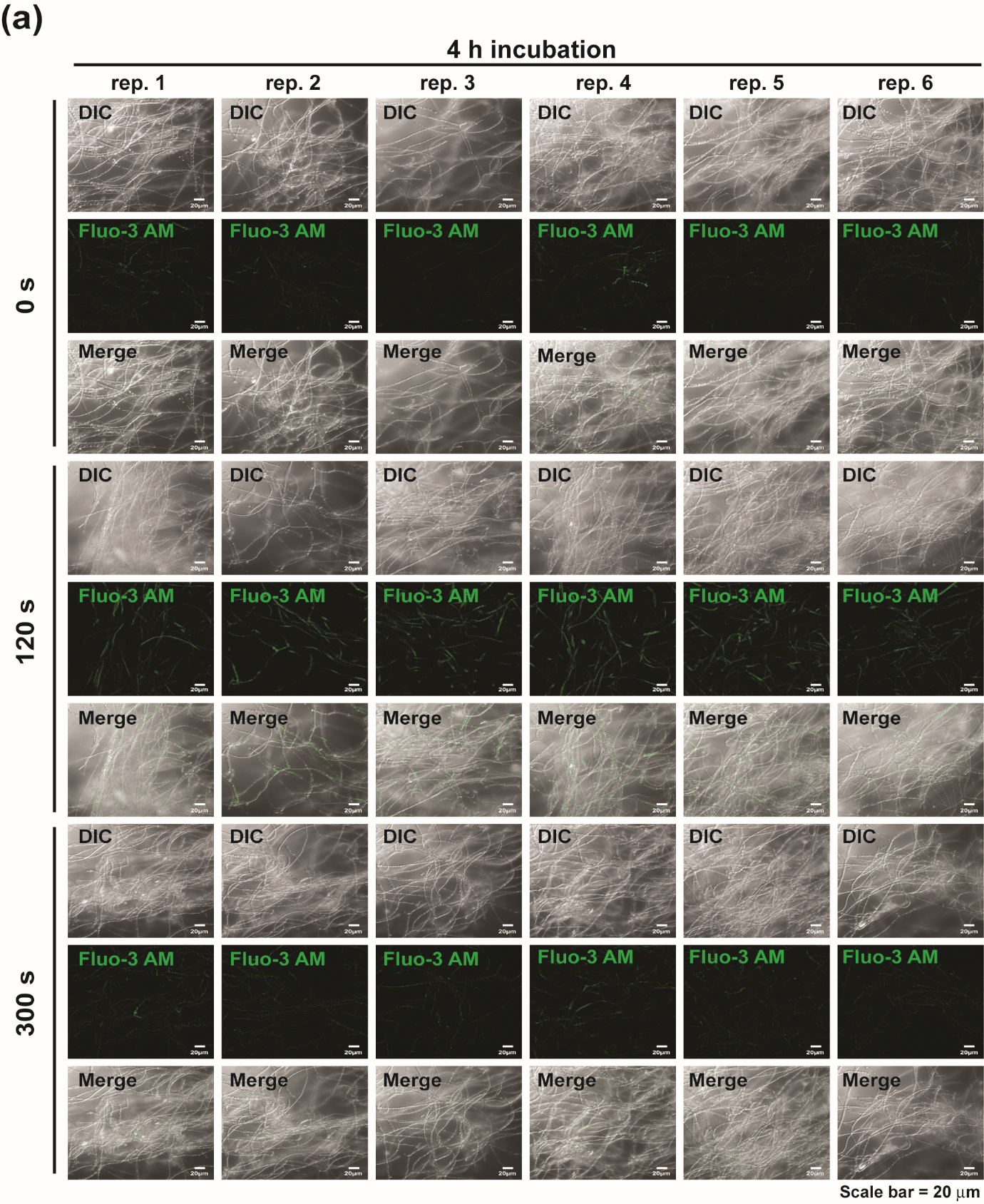


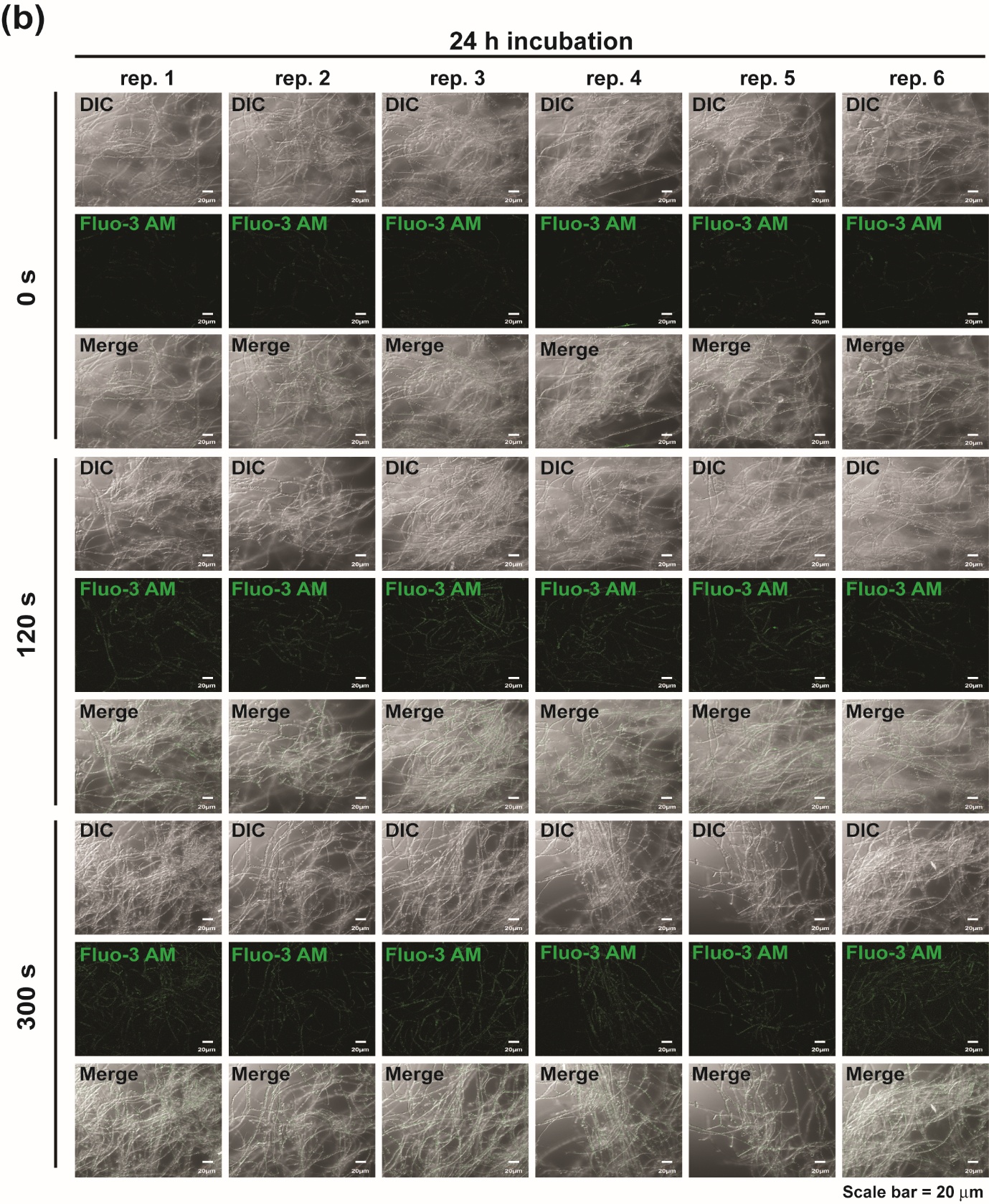


**
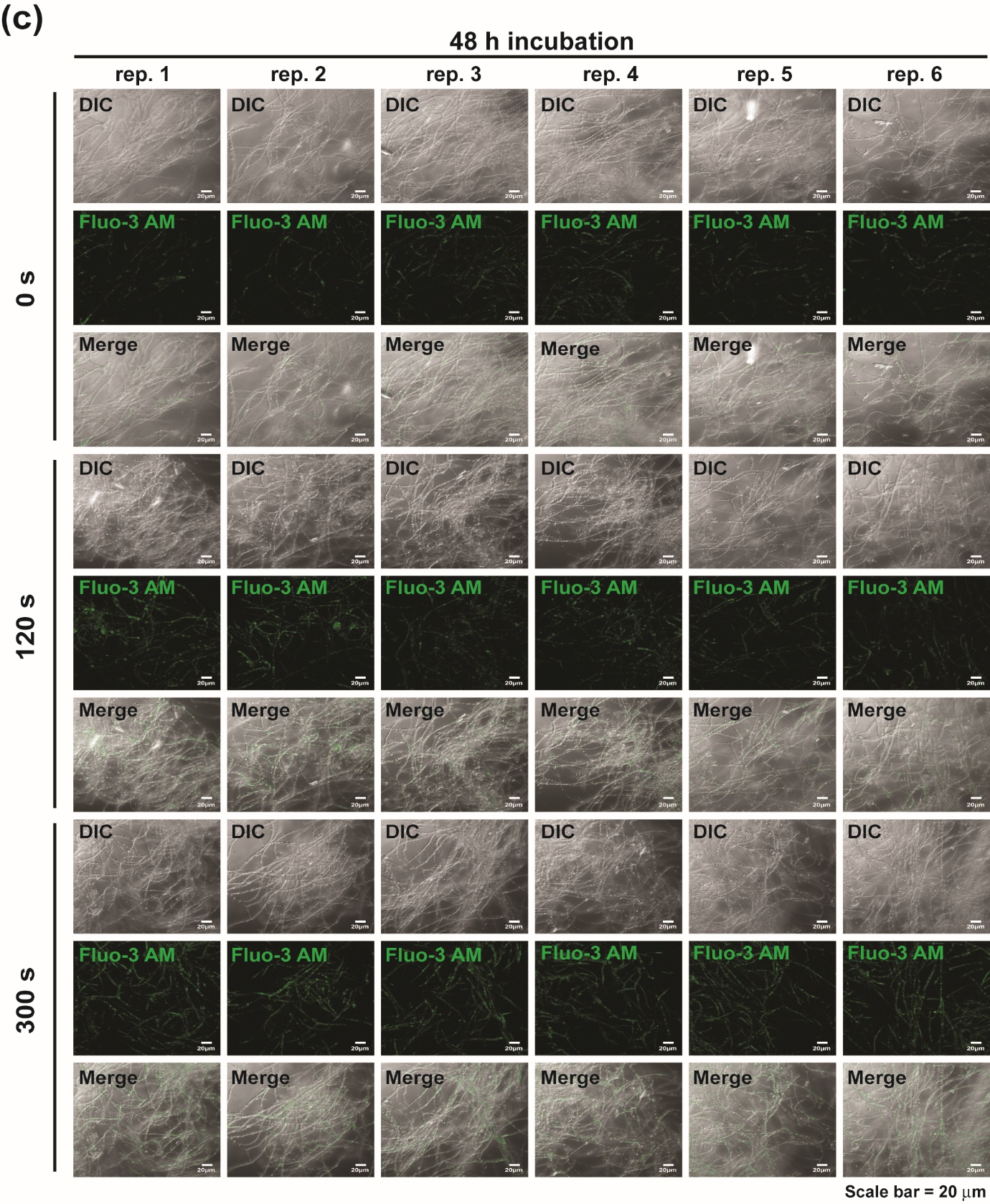
**

Supplementary Figure S4. Analysis for intracellular Ca^2+^ accumulation after plasma treatment. Hyphae were stained with Fluo-3 AM (green fluorescence) after 4h (a), 24h (b) and 48h (c) of plasma treatment. Pictures of fungal hyphae were taken in 6 different areas. DIC; Differential Interference Contrast, Fluo-3 AM; fluorescence, Merge; combined image of DIC and fluorescence.


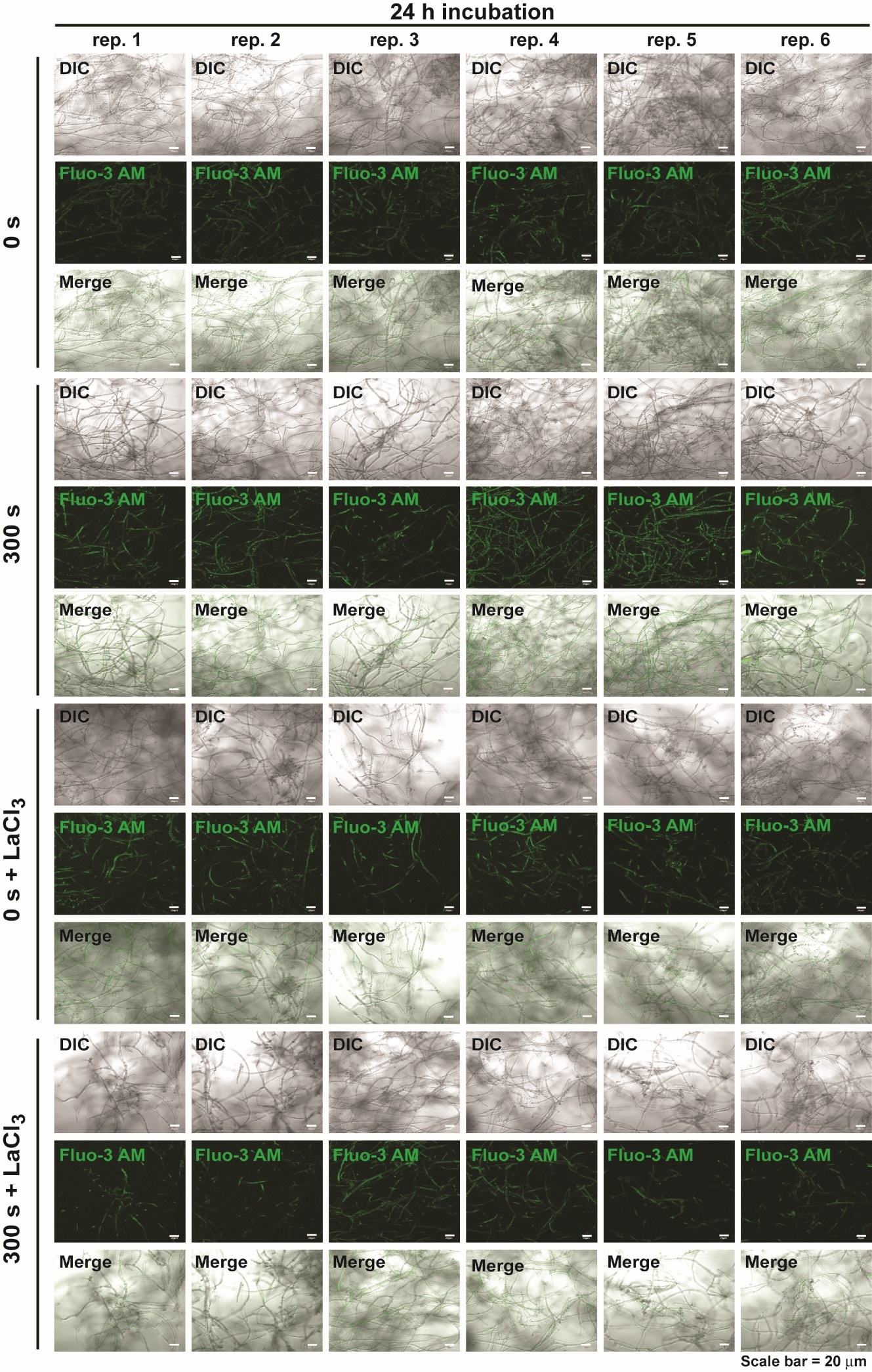


Supplementary Figure S5. Intracellular Ca^2+^ level in plasma-treated fungal hyphae under the condition of inhibition of Ca^2+^ channel. Ca^2+^ stained with Fluo-3 AM in wild type (FGSC4200) fungal hyphae incubated for 24 h after treatment with MS-DBD plasma for 0s or 300s, followed by addition of avicel supplemented with LaC1_3_ (Ca^2+^ channel inhibitor, 5 mM) or none. Pictures of fungal hyphae were taken in 6 different areas. DIC; Differential Interference Contrast, DAF-FM DA; fluorescence, Merge; combined image of DIC and fluorescence.


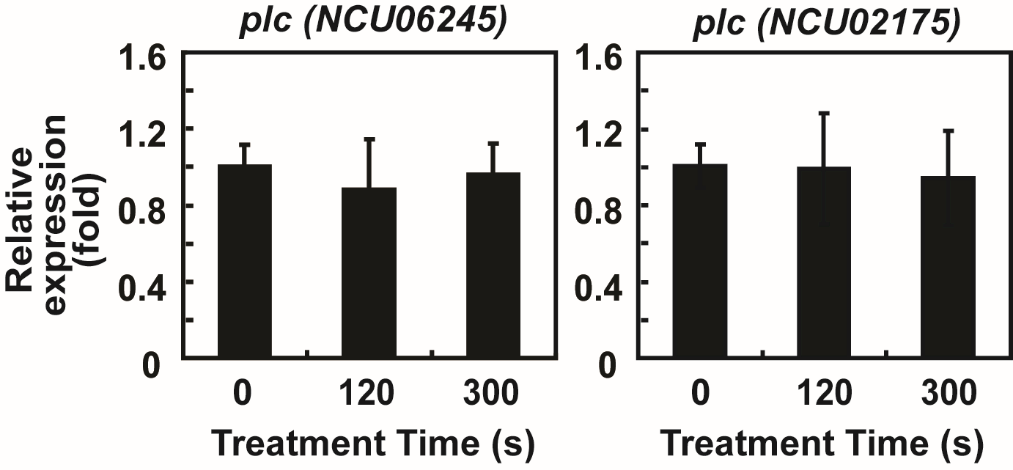


Supplementary Figure S6. Level of mRNA of *plc* genes (NCU06245 and NCU02175) in fungal hyphae. Fungal hyphae were grown in avicel media for 4 h after plasma treatment. Relative mRNA level compared to that of no plasma treated group was calculated. Each value is the average of 9 replicate measurements (three independent biological replicates and three technical replicates).


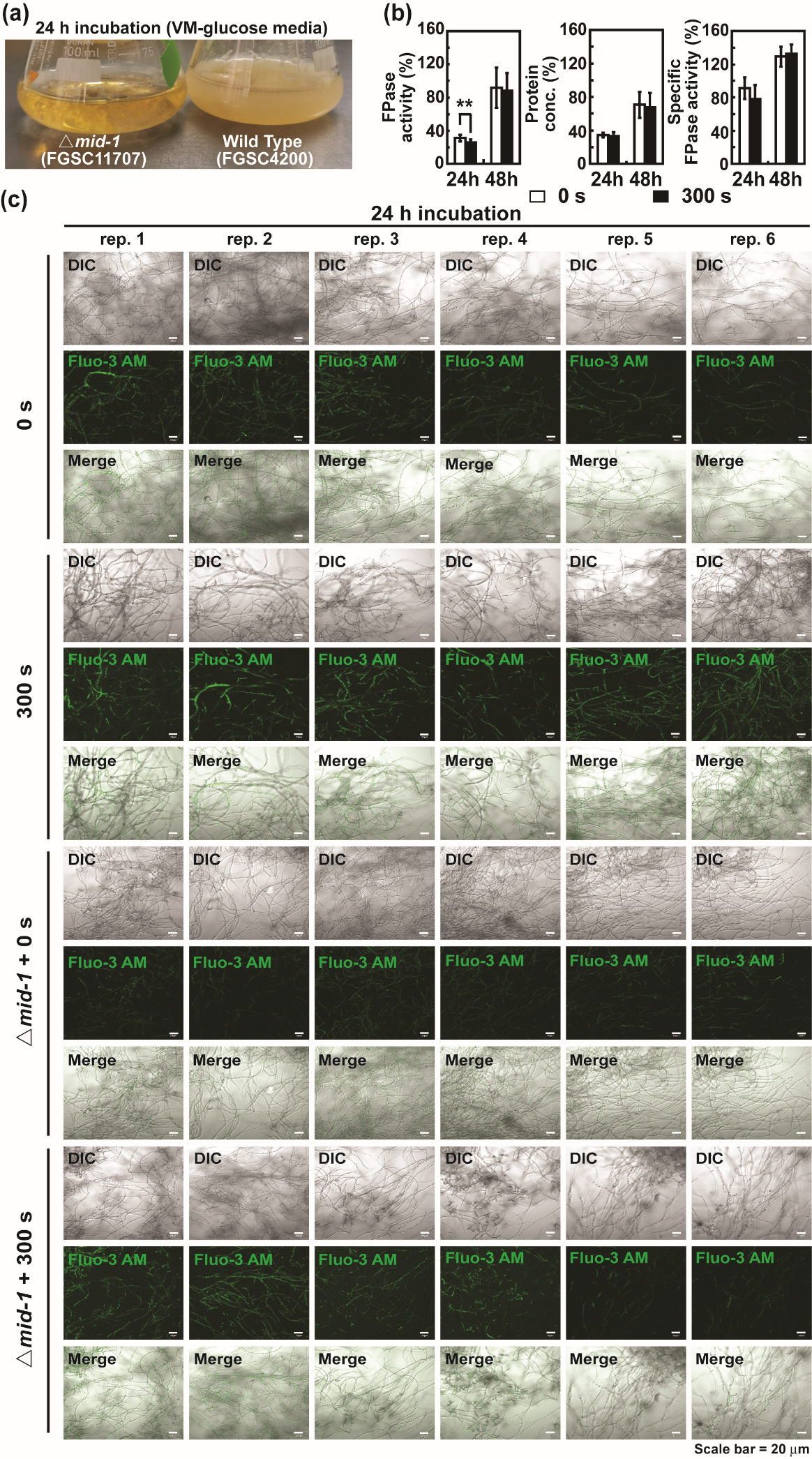


Supplementary Figure S7. **Effects of deletion of** Ca^2+^ channel on cellulases production and intracellular Ca^2+^ level. **(a)** Growth of knockout mutant of Ca^2+^ channel-encoded gene *mid-1* (FGSC11707) in VM-glucose media. **(b)** FPase activity, total protein concentration, and specific FPase activity in media 24 and 48 h after *mid-1* mutant was treated with MS-DBD plasma and none, followed by addition of avicel. Each value represents the relative percentage compared to the value of wild type treated with none (control), averaging 9 replicate measurements (three independent biological replicates and three technical replicates): ** *p* < 0.01 as determined by Student’s *t*-test. **(c)** Ca^2+^ stained with Fluo-3 AM in hyphae of wild type and *mid-1* deletion mutant (FGSC11707) incubated for 24 h after treatment with MS-DBD plasma for 0s or 300s, followed by addition of avicel. Pictures of fungal hyphae were taken in 6 different areas. DIC: differential interference contrast, Fluo-3 AM: fluorescence, Merge: combined image of DIC and fluorescence.


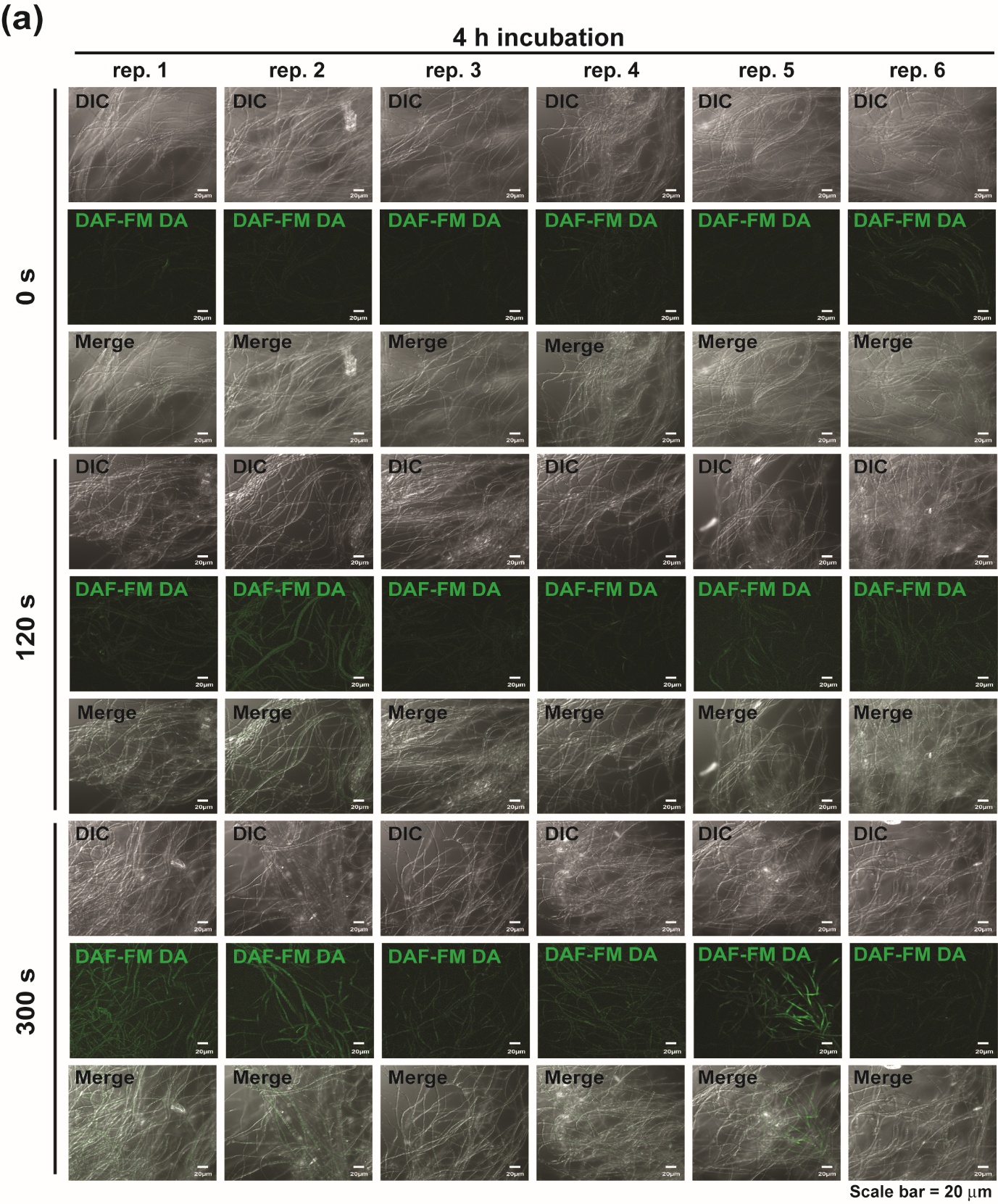


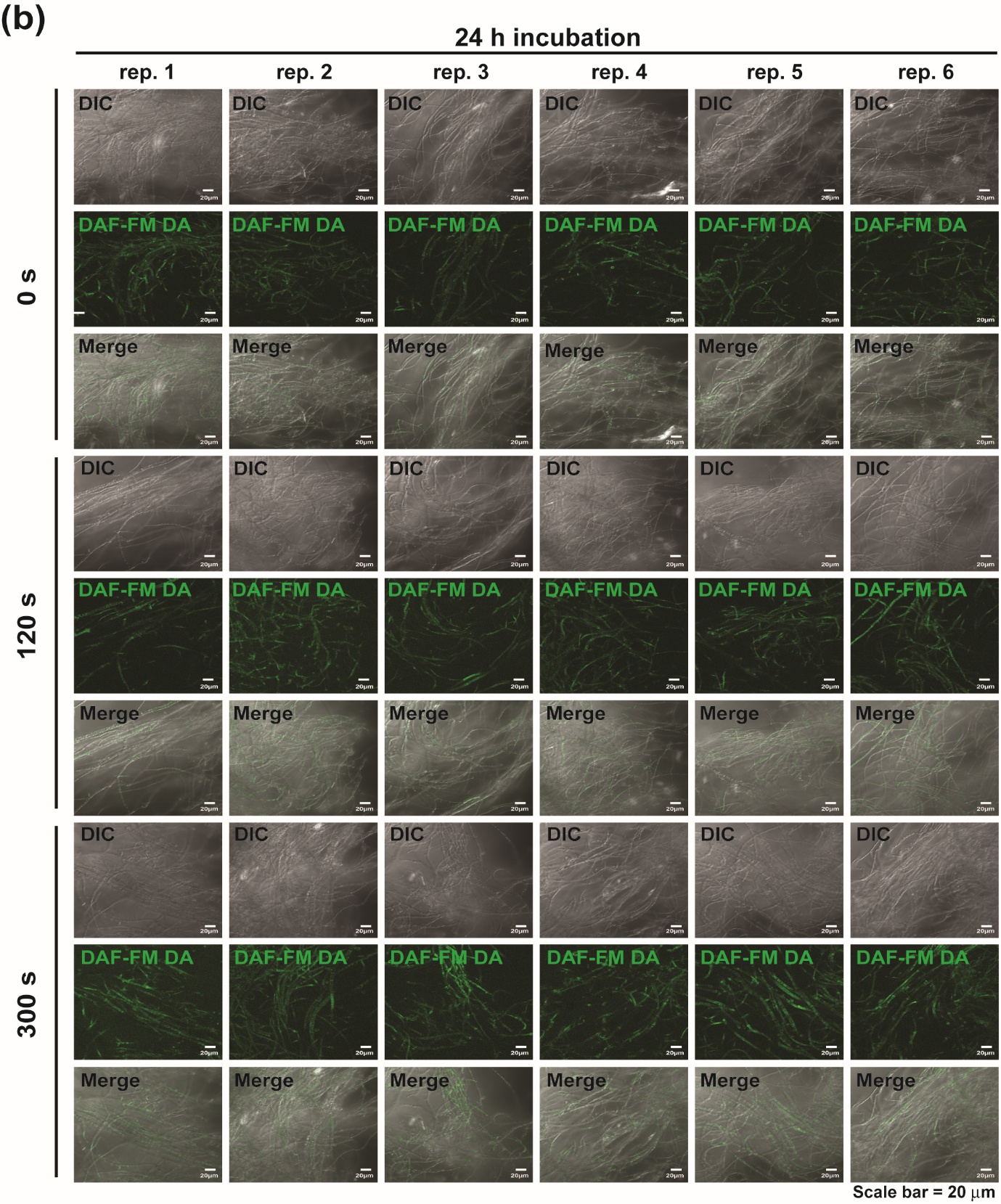


**
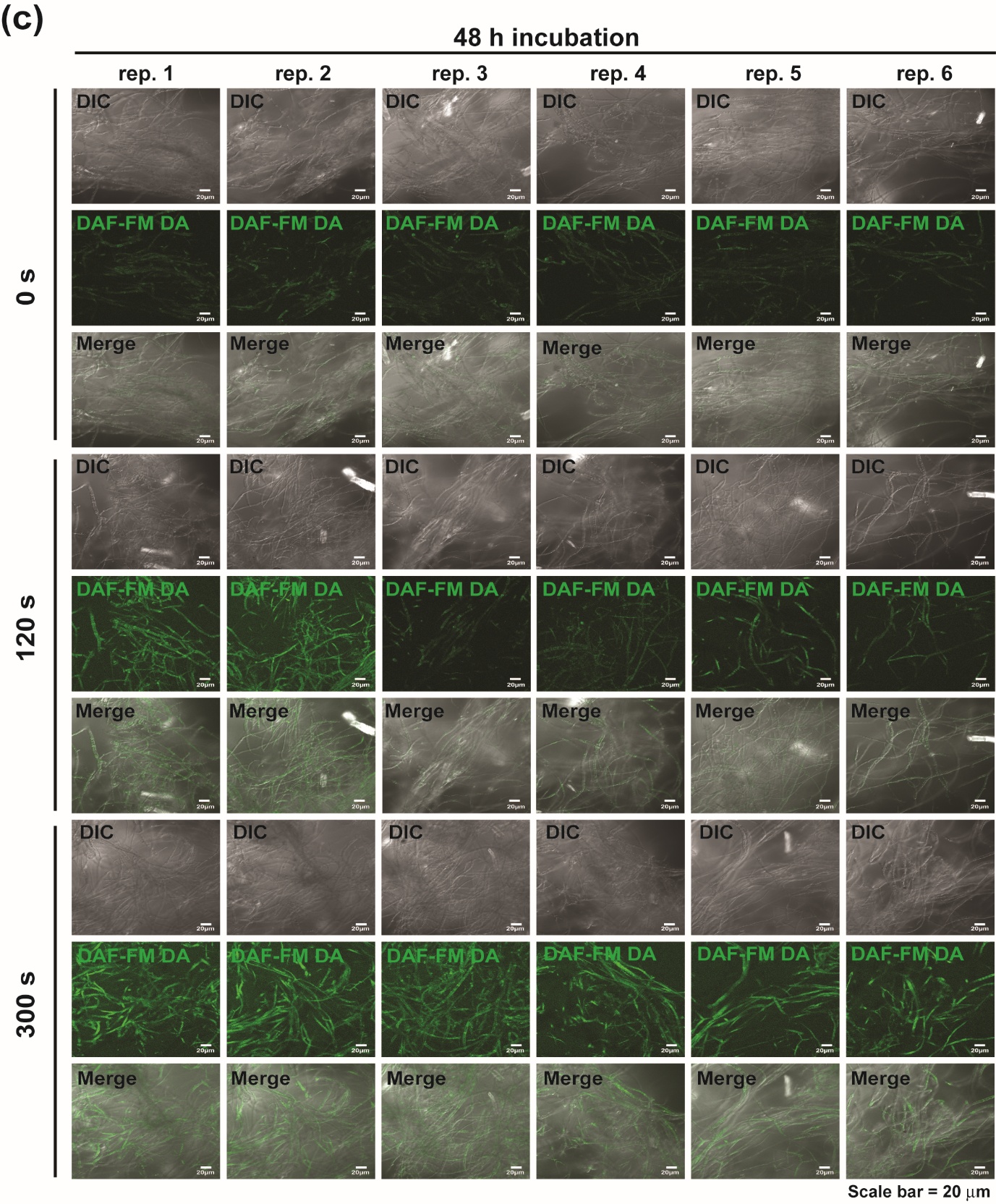
**

Supplementary Figure S8. Intracellular **NO** level in *N. crassa* hyphae after MS-DBD plasma treatment. Fungal hyphae were stained with DAF-FM DA (green fluorescence) after 4 h (a), 24 h (b) and 48 h (c) of plasma treatment. Pictures of fungal hyphae were taken in 6 different areas. DIC; Differential Interference Contrast, DAF-FM DA; fluorescence, Merge; combined image of DIC and fluorescence.


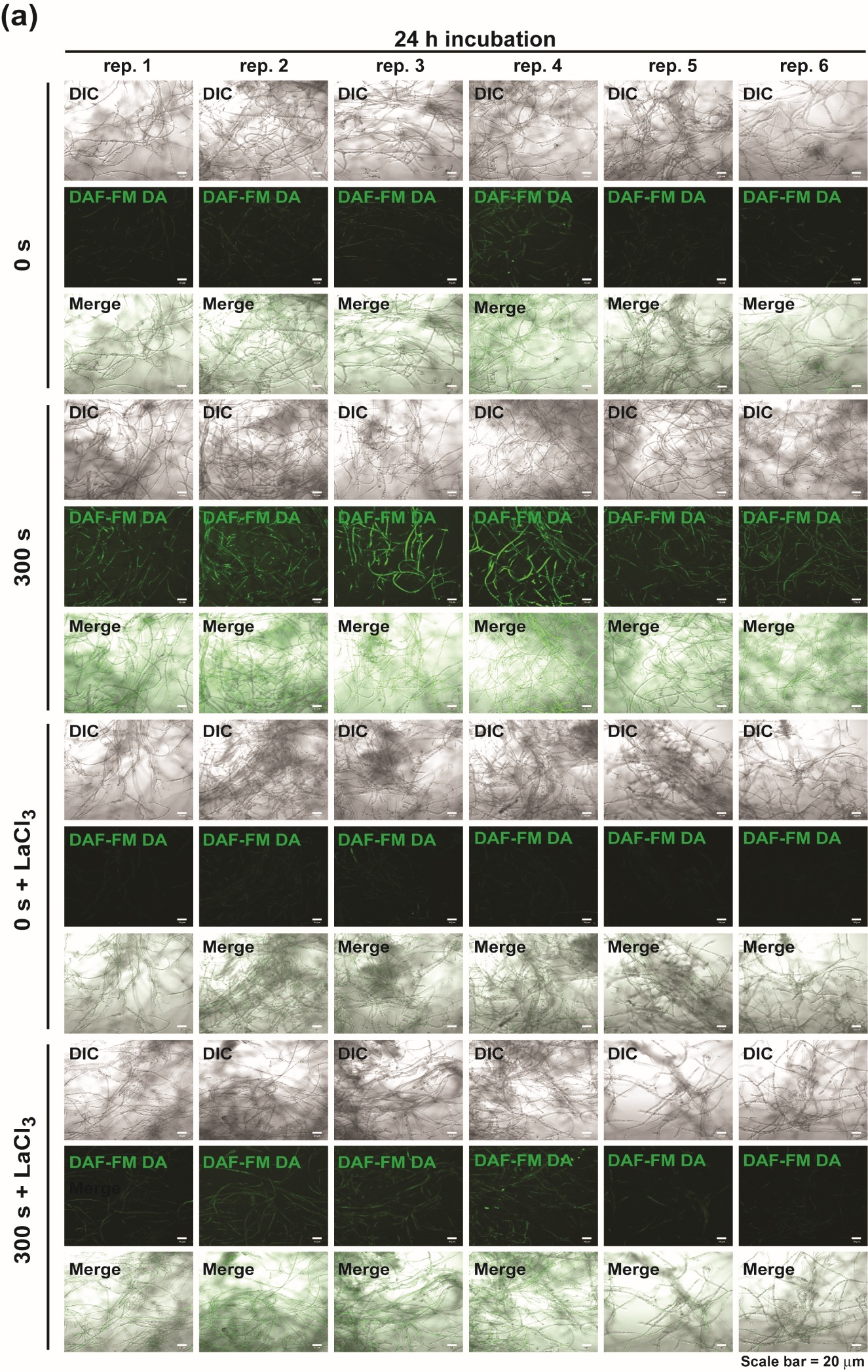


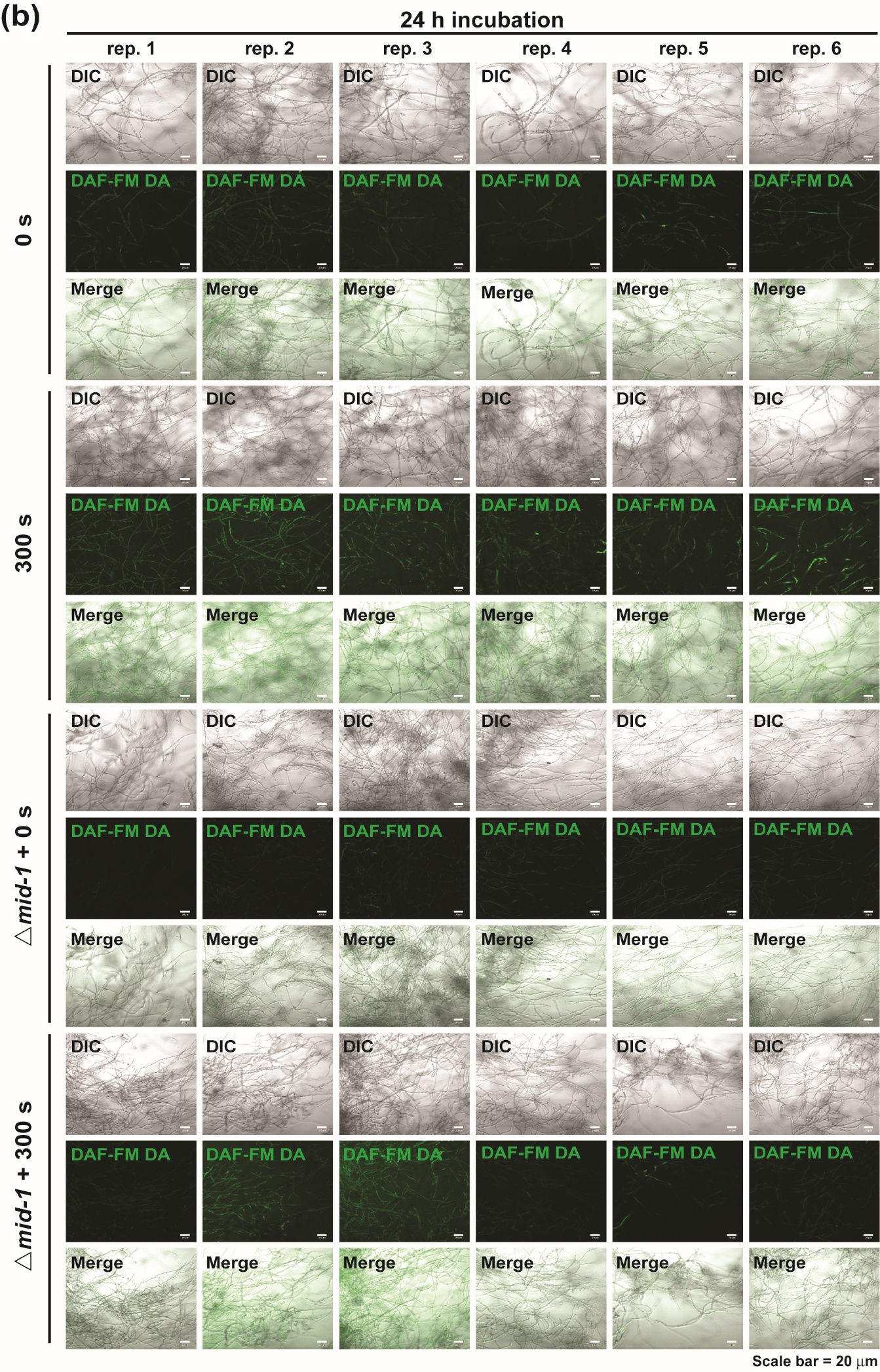


Supplementary Figure S9. Intracellular NO level in LaC1_3_-treated wild type (FGSC4200) **(a) and** *mid-1* mutant (FGSC11707) **(b) hyphae**. The hyphae were cultured in avicel media with or without LaC1_3_, respectively, then recovered after 24 h of incubation, and stained with DAF-FM DA (green fluorescence). Pictures of fungal hyphae were taken in 6 different areas. DIC; Differential Interference Contrast, DAF-FM DA; fluorescence, Merge; combined image of DIC and fluorescence.


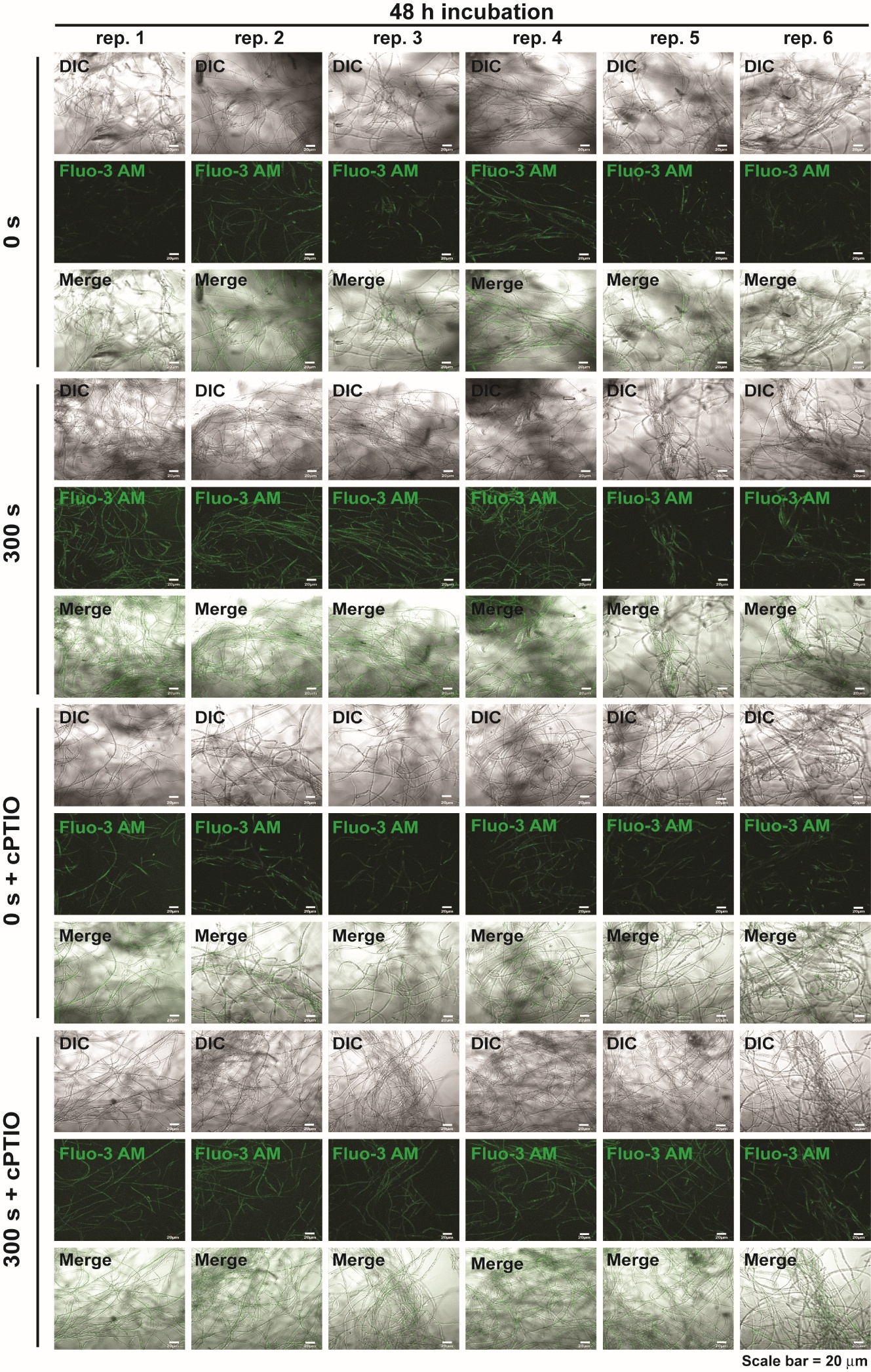


Supplementary Figure S10. Intracellular Ca^2+^ levels in hyphae after 48 h of plasma and cPTIO treatment. Hyphae were stained with Fluo-3 AM (green fluorescence) after 48h of incubation from plasma treatment in avicel media supplemented with cPTIO or none. Pictures of fungal hyphae were taken in 6 different areas. DIC; Differential Interference Contrast, Fluo-3 AM; fluorescence, Merge; combined image of DIC and fluorescence.


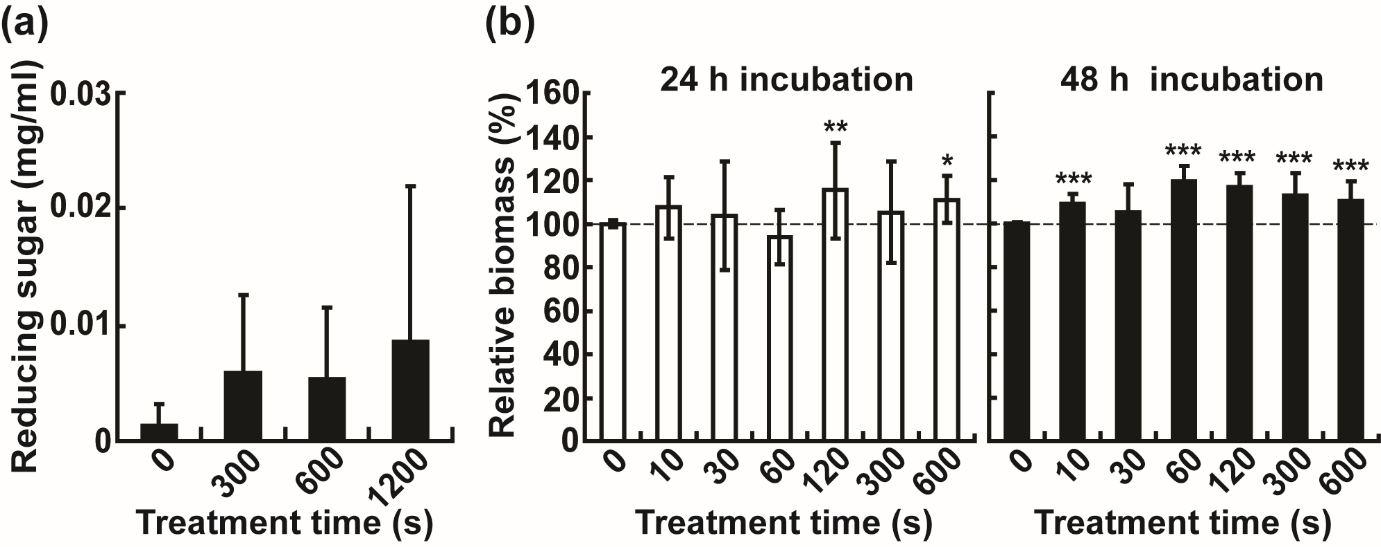


Supplementary Figure S11. Effects of MS-DBD plasma treatment on avicel degradation and fungal hyphal growth in media. **(a)** Level of total reducing sugars produced in avicel medium after MS-DBD plasma treatment. (b) Relative percentage of dry weight (biomass) of fungal mycelia compared to those of non-treated groups grown in glucose media after MS-DBD plasma treatment. Fungal mycelia were harvested after 24 h and 48 h of incubation. Each value is the mean of 6 or 9 replicate measurements (three independent biological replicates and two-three technical replicates): * p < 0.05, ** p < 0.01, *** *p* < 0.001 as determined by Student’s *t*-test (control vs. treatment).
